# Supplementary material for: Epithelial cell competition is promoted by signaling from immune cells
Source: Nat Commun. 2025 Apr 19;16:3710. doi: 10.1038/s41467-025-59130-5 (PMC12008283; doi:10.1038/s41467-025-59130-5)
Supplement: Supplementary file 1 — Supplementary Information [file 41467_2025_59130_MOESM1_ESM.pdf]

SUPPLEMENTARY FILES for
Epithelial cell competition is promoted by signaling from immune cells

Yilun Zhu, Zeba Wunderlich, and Arthur D. Lander

Supplementary Figures S1 – S22

Supplementary Tables S1 – S2

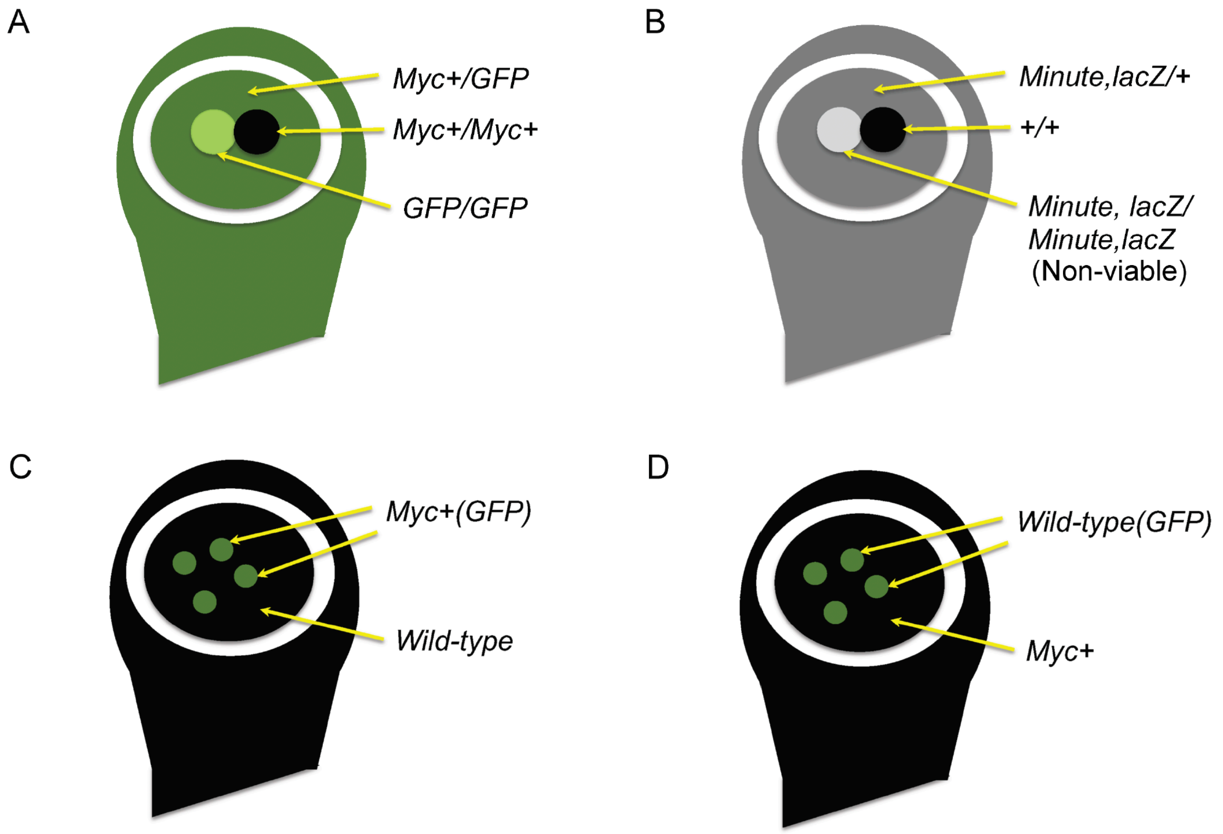

Figure S1. Clonal induction methods. The genotypes and fluorescent marking of clones used to elicit cell competition in this study are shown: (**A**) *dMyc* twin-spot clones. Twin-spot mosaic clones in a background carrying an extra copy of *dMyc* (green), were generated that either have two extra copies of *dMyc* (black, no GFP) or no extra copies of *dMyc* (bright green, 2x GFP). (**B**) *Minute* twin-spot clones. Twin-spot mosaic clones in a background carrying a copy of *Minute* (gray, one copy of LacZ), either have two copies of *Minute* (bright gray; two copies of LacZ) or no copies of *Minute* (black, no LacZ). Typically, cells with two Minute alleles do not survive. (**C**) *dMyc* flip-out clones. Flip-out mosaic clones carrying an extra copy of *dMyc* and GFP (green) in a wild-type background (black). (**D**) Wild-type flip-out clones. Flip-out mosaic clones carrying a copy of GFP (green) in a *dMyc* background (black).

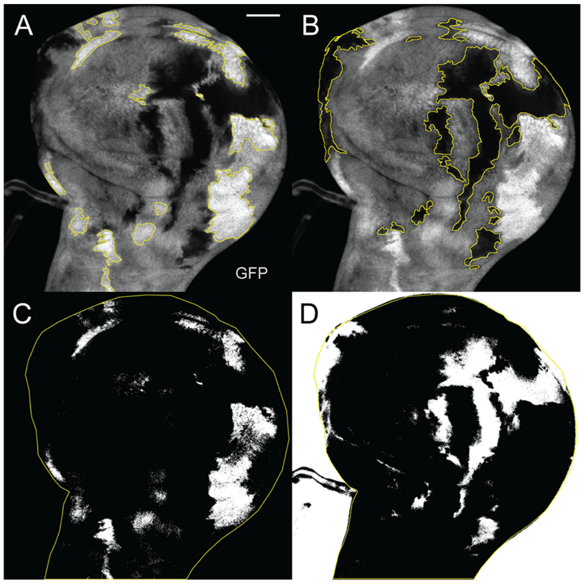

E

Myc clones	Area	Pixel
Area ratio (dark/bright)	1.5568	1.60211

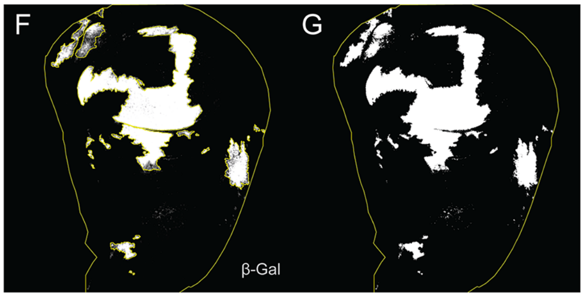

H

Minute clones	Area	Pixel
Area ratio (brihgt/dark)	0.2074	0.20756

Figure S2. Quantification of cell competition. (A-E) Comparison of quantification of *dMyc*-induced cell competition using two different methods. Super-competition is quantified by measuring the area ratio between twin-spot clones—GFP-negative cells (dark) and GFP-double-positive cells (bright). The first method to calculate the area ratio is a traditional protocol in which the dark clone area (B) is divided by the bright clone area (A). The second method, we used in this study, is to compare pixel percentages between dark clones (D) and bright clones (C). In (C), the brightness of the image is adjusted to a specific threshold where only bright clones can be observed, then the number of pixels above this brightness level is considered as the number of pixels in bright clones and then is divided by the total number of pixels in the whole wing disc to yield a pixel percentage of bright clones. In (D), above steps are repeated after inverting the image to obtain a pixel percentage of dark clones. Comparison of area ratios between dark and bright clones acquired by these two methods (E). **(F-H)** Comparison of quantification of *Minute*-induced cell competition utilizing two different methods. Classic cell competition quantified by measuring the area ratio between *Minute* (bright, carrying *arm-lacZ*) and wild-type (dark, no *arm-lacZ*) cells. In (F), the bright clone area is divided by the dark clone area to yield the area ratio. In (G), the number of pixels in bright clones is acquired as in C, but then divided by the number of rest pixels in the disc to yield the area ratio. Comparison of area ratios between bright and dark clones acquired by these two methods (H). Bar = 50 μm.

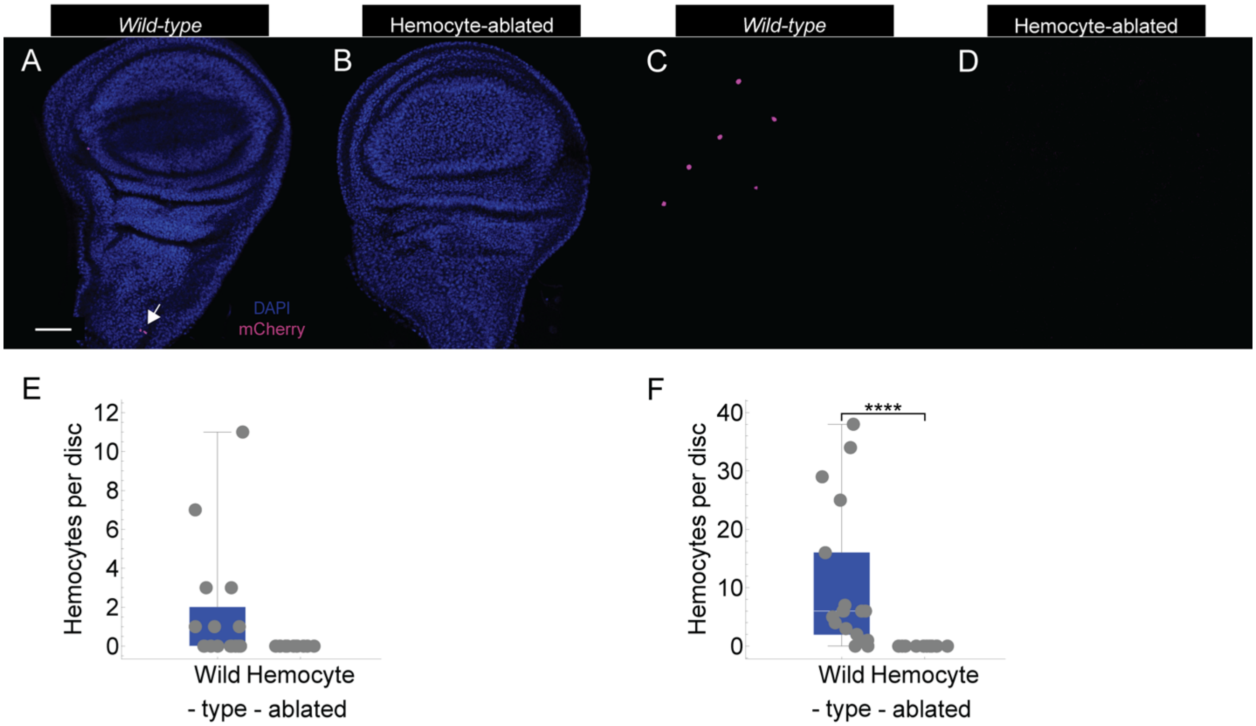

Figure S3. Efficiency of hemocyte ablation. (**A-B**) Representative wild-type (A) and hemocyte-ablated (B), wing discs. Discs are stained with DAPI and hemocytes visualized using *srpHemo-H2A.3XmCherry*. (**C-D**) Representative wing discs of genotypes in panels A and B, fixed but imaged immediately without washing (and therefore also without DAPI). (**E-F**) Hemocytes per disc for the genotypes in A-B (panel E) or C-D (panel F). n = 16, 10, 18, and 12 for the genotypes in (A), (B), (C), and (D), respectively. Significance was tested using the Mann-Whitney U test (****=P<0.0001). Scale bar = 50 μm. Source data are provided as a Source Data file.

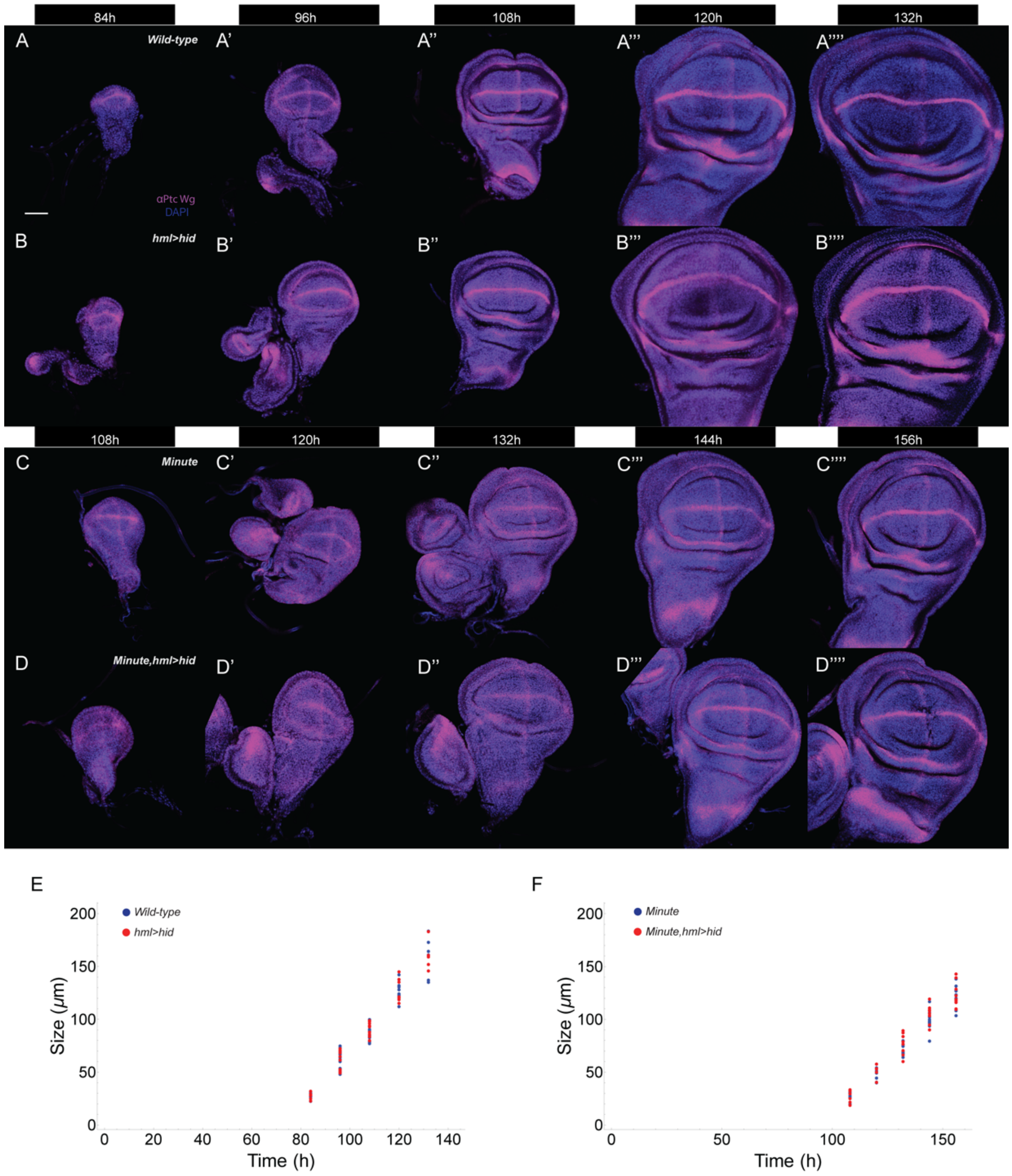

Figure S4. Hemocyte ablation does not delay normal development. (**A-B''''**) Representative images of wild-type (A-A'''') and hemocyte-ablated (B-B'''') wing discs at various times after egg laying (84 h, 96 h, 108 h, 120 h and 132 h AEL). (**E**) Quantification of the size of posterior compartments of genotypes in (A-B''''). The compartment size was quantified by measuring the length of a segmented line tracing Wg antibody staining at the D/V compartment boundary, starting at the A/P compartment boundary, and ending at the edge of the wing pouch edge. *n* = 11, 5, 18, 10, 15, 11, 12, 7, 6, and 5 for the genotypes in (A), (B), (A'), (B'), (A''), (B''), (A'''), (B'''), (A''''), and (B''''), respectively. (**C-D''''**) Representative images of wing discs carrying a copy of *Minute* mutation in a wild-type background (C-C'''') or a background in which hemocytes were ablated (D-D'''') at various times after egg laying (108 h, 120 h, 132 h, 144 h and 156 h AEL). (**F**) Quantification of the size of posterior compartments of genotypes in (C-D''''). *n* = 8, 6, 8, 5, 10, 8, 12, 9, 8, and 9 for the genotypes in (C), (D), (C'), (D'), (C''), (D''), (C'''), (D'''), (C''''), and (D''''), respectively. Scale bar = 50 μm. Source data are provided as a Source Data file.

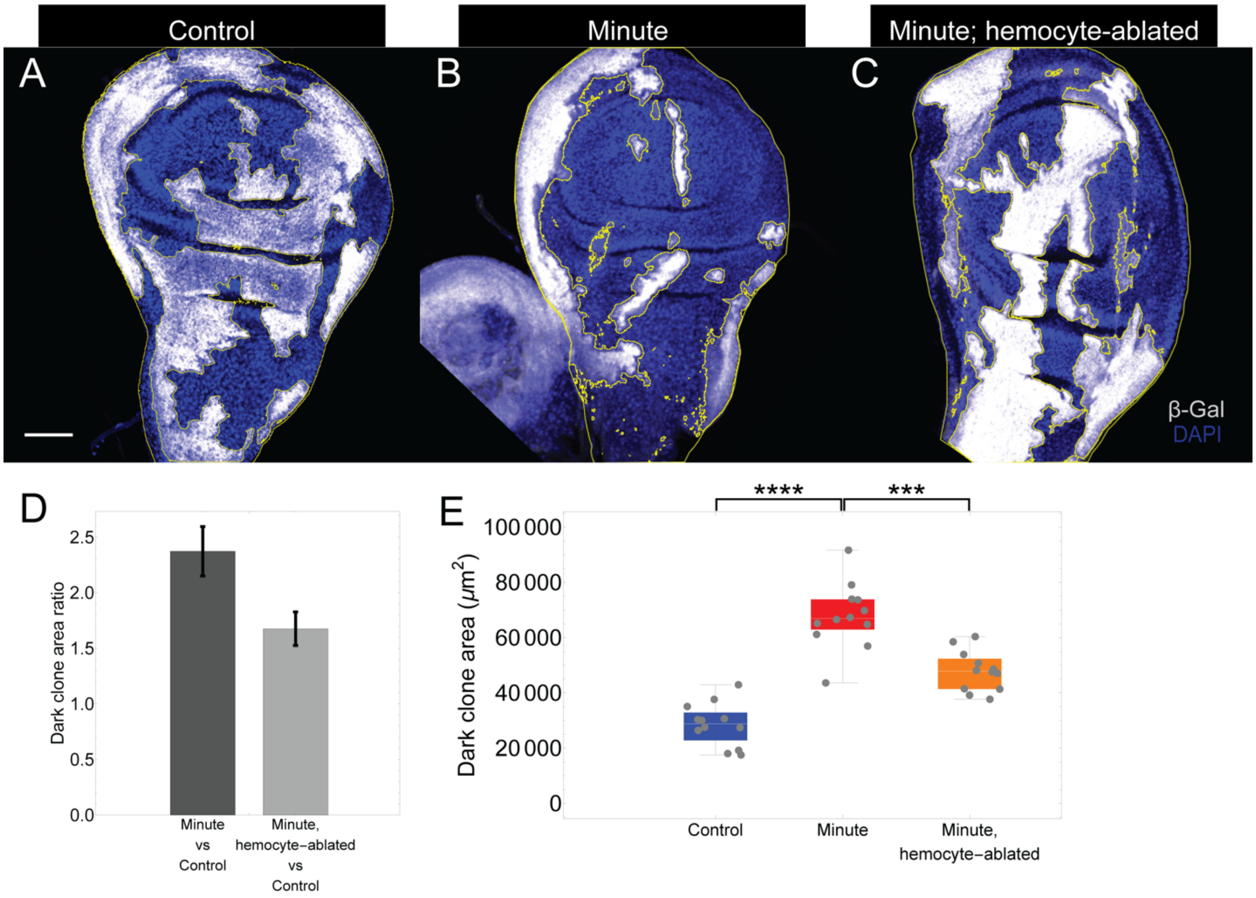

Figure S5. Alternate analysis of minute clones, related to Figure 1. (A-C) Representative wing discs in Fig.1D-F with clones outlined, respectively. **(D)** Ratio of average dark clone area in wing discs of (B) to (A), and ratio of average dark clone area in wing discs of (C) to (A). Error bars = ± SEM. **(E)** Calculated total area of dark clones in each wing disc of genotypes in (A-C). *n* = 13, 15, and 12 for the genotypes in (A), (B), and (C), respectively. Statistical significance was tested using the Mann-Whitney U test (***=P<0.001; ****=P<0.0001). The scale bar is 50 µm. Source data are provided as a Source Data file.

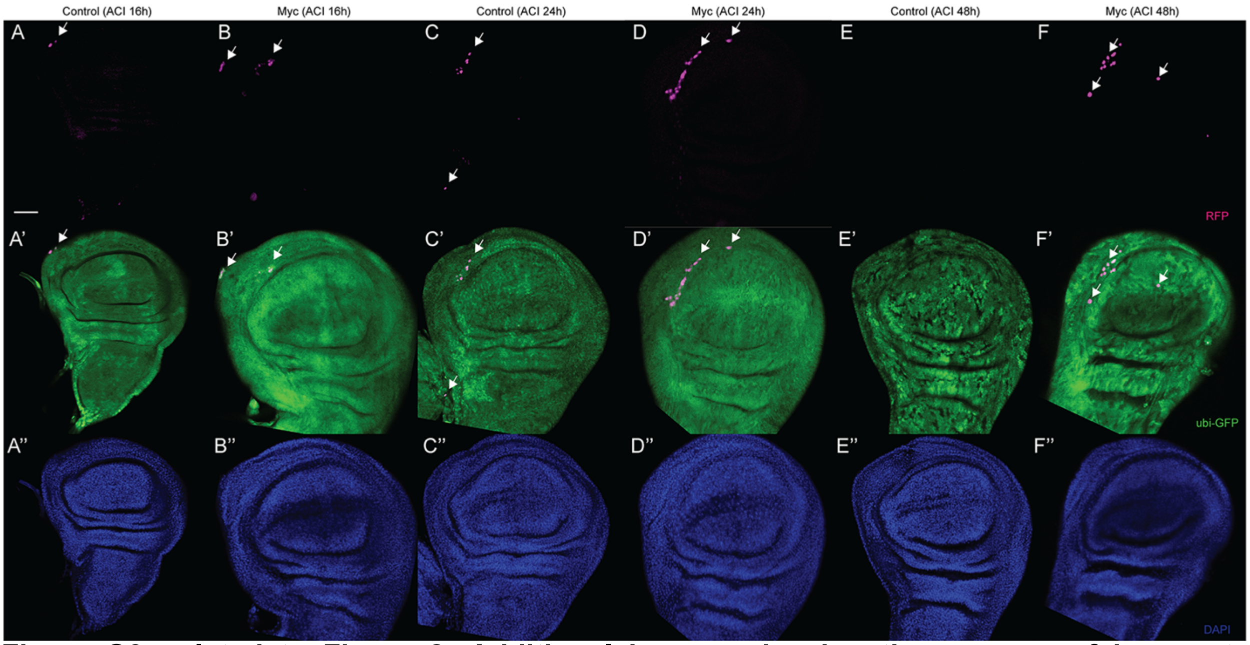

Figure S6, related to Figure 2. Additional images showing time course of hemocyte recruitment after induction of cell competition. (A-F'') Representative wing discs carrying control and *dMyc* clones were collected at defined time periods—16, 24 or 48 h ACI, with hemocytes labeled by RFP (A-F', pointed out by white arrows), stained with DAPI (A''-F''). In control groups, GFP-negative, GFP-single-positive, and GFP-double-positive cells are all wildtype. In experimental groups, GFP-negative cells have two extra copies of *dMyc*, GFP-single-positive cells have one extra copy of *dMyc* and GFP-double-positive cells have no extra copy of *dMyc*. The scale bar is 50 μm.

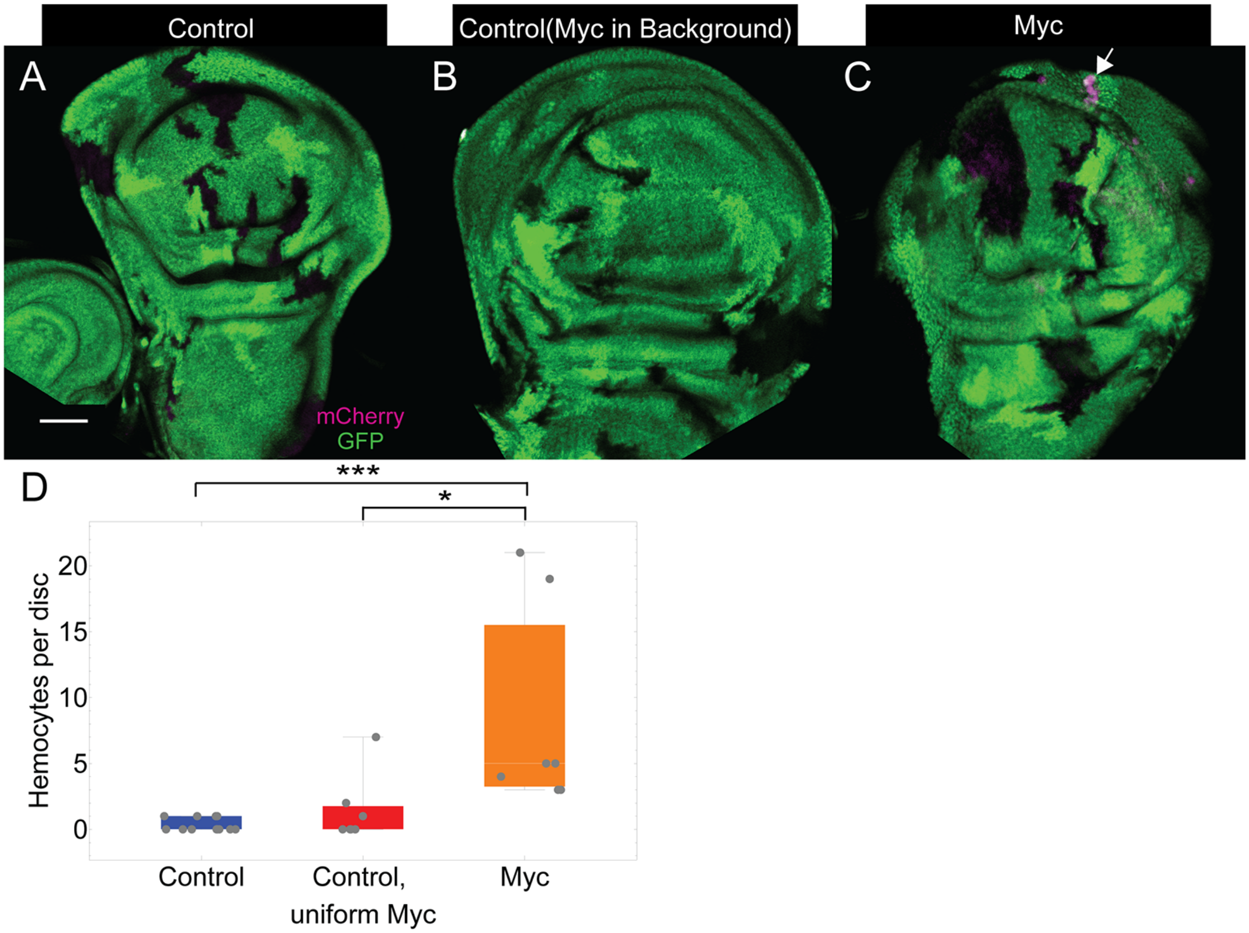

Figure S7, related to Figure 2. Supporting information for hemocyte recruitment by cell competition. (A-C) Representative wing discs carrying control clones in a wild-type background (A), neutral clones (GFP only) in a 1x-*dMyc* background (B) and *dMyc* clones (C) with hemocytes labeled by mCherry, 72 h ACI. **(D)** Quantification of the number of hemocytes in wing discs of the genotypes in (A-C). n = 12, 7, and 7 for the genotypes in (A), (B), and (C), respectively. Statistical significance was tested using the Mann-Whitney U test (*=P<0.05; ***=P<0.001). Scale bar, 50 µm. Source data are provided as a Source Data file.

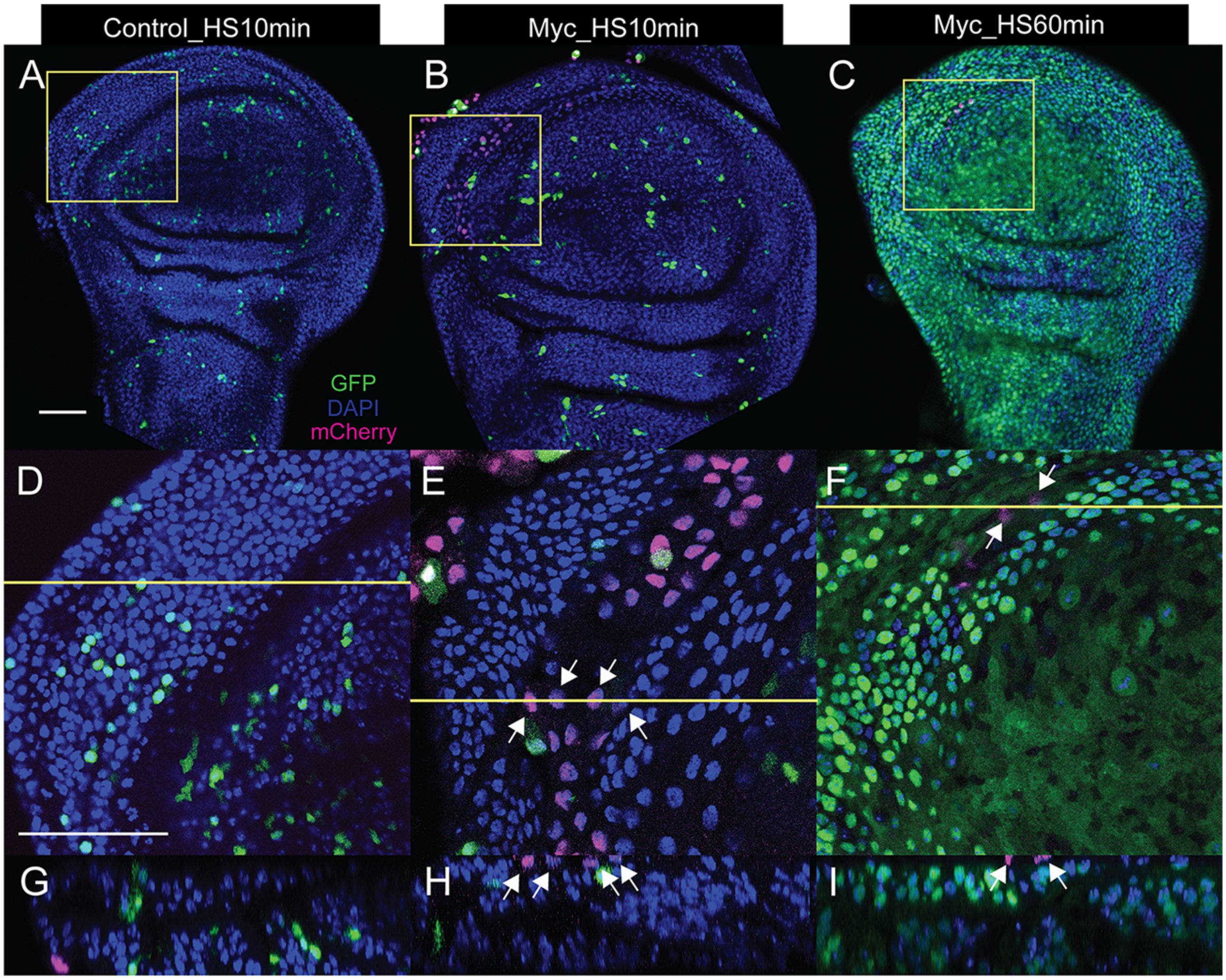

J

srpmCherry(II)	Peripodial	Apical	Basal
Control_HS10min	0.41	0.03	0.56
Myc_HS10min	0.57	0	0.43
Myc_HS60min	0.46	0	0.54

K

srpmCherry(III)	Peripodial	Apical	Basal
Control_HS10min	0.15	0	0.85
Myc_HS10min	0.6	0	0.4
Myc_HS60min	0.43	0	0.57

Figure S8, related to Figure 2. Recruited hemocytes are mainly located at peripodial epithelial and basal surfaces. (A-I) Representative wing discs carrying flip-out mosaic clones with hemocytes labeled by mCherry, stained with DAPI generated as in Fig. 4A-C, 14 h ACI. (D-F) are enlargements of rectangles in (A-C). Corresponding cross-sections of wing discs extracted along the yellow lines in D-F (G-I). **(J-K)** The proportion of hemocytes present at the peripodial epithelium, apical surface, or basal surface of wing discs of the genotypes in Fig. S14A-C (J) or Fig. 4A-C (K), respectively. *n* = 15, 12, and 19 for the genotypes in (J), and *n* = 7, 9, 12 in for the genotypes in (K), respectively. Bars represent 50 μm (A-C) and 10 μm (D-F). Source data are provided as a Source Data file.

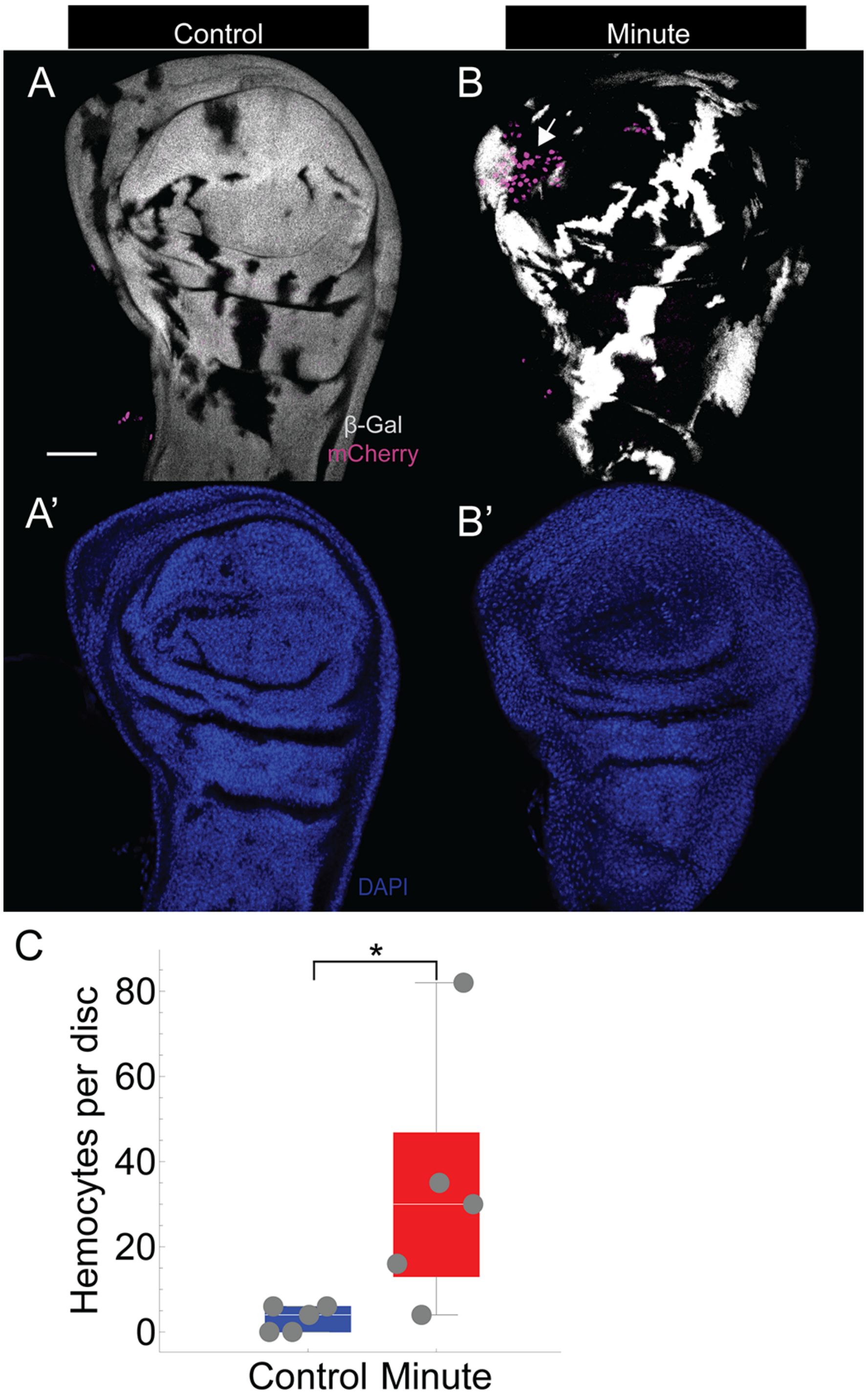

Figure S9, related to Figure 2. Hemocyte recruitment in *Minute*-induced cell competition. (**A-B**) Representative wing imaginal discs carrying control (A) and *Minute* clones (B) visualized by staining with anti-β-galactosidase antibodies, 96 h ACI; hemocytes visualized using *srpHemo-H2A.3XmCherry*. (**A'-B'**) Wing discs in (A-B) were stained with DAPI. (**C**) Number of hemocytes per disc for the genotypes in A and B. $n = 5$ in each case. Statistical significance was tested using the Mann-Whitney U test (*=$P<0.05$). Scale bar = 50 µm. Source data are provided as a Source Data file.

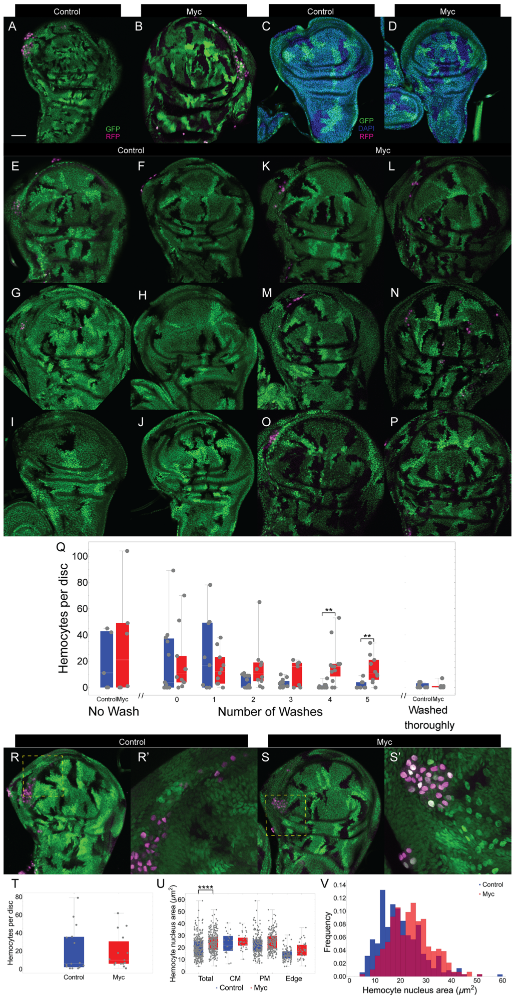

Figure S10, related to Figure 2. Differential effects of washing on hemocytes numbers reveals the influence of cell competition on hemocyte attachment to wing discs. (A-D) Representative wing discs carrying either control clones in a wild-type background (A,C) or *dMyc* clones (B,D). Hemocytes are marked by RFP. In panels A and B, discs were fixed as usual, but not washed. In C and D, discs were fixed for only 20 min, followed by seven 10-min washes, DAPI staining, and 5 additional washes[57]. **(E-P)** Representative discs carrying control clones (E-J) and *dMyc* clones (K-P) generated as in (A-B) and washed 0, 1, 2, 3, 4 or 5 times after fixation, respectively. (**Q**) Quantification of the number of hemocytes in wing discs of the experiments in (A-P). *n* = 5, 5, 8, 7, 11, 9, 15, 12, 12, 9, 10, 10, 10, 7, 12, and 10 discs for the experiments illustrated in A-P, respectively. **(R-S')** Representative wing discs carrying control clones (R) and *dMyc* clones (S) generated and processed exactly as in (A-B). R' and S' are higher magnification views of the outlined areas. *n* = 15 for each of the genotypes in (R) and (S), respectively. (**T**) Numbers of hemocytes in discs from the experiments shown in R (control, blue bars) and S (dMyc, red bars). (**U**) Quantification of hemocyte nuclear area in discs from the experiments shown in panels R and S. *n* = 317 and 284 for the two genotypes, respectively. Observations were subdivided into groups based on the part of the disc examined (Columnar membrane, CM; Peripodial membrane, PM; Edge). Statistical significance was tested using the Mann-Whitney U test (**=*P*<0.01; ****=*P*<0.0001). (**V**) Distributions of hemocyte nuclear areas in wing discs from panel U. Areas from control discs are in blue; from dMyc discs are in red; dark maroon marks the overlap between the two histograms. Scale bar, 50 µm. Source data are provided as a Source Data file.

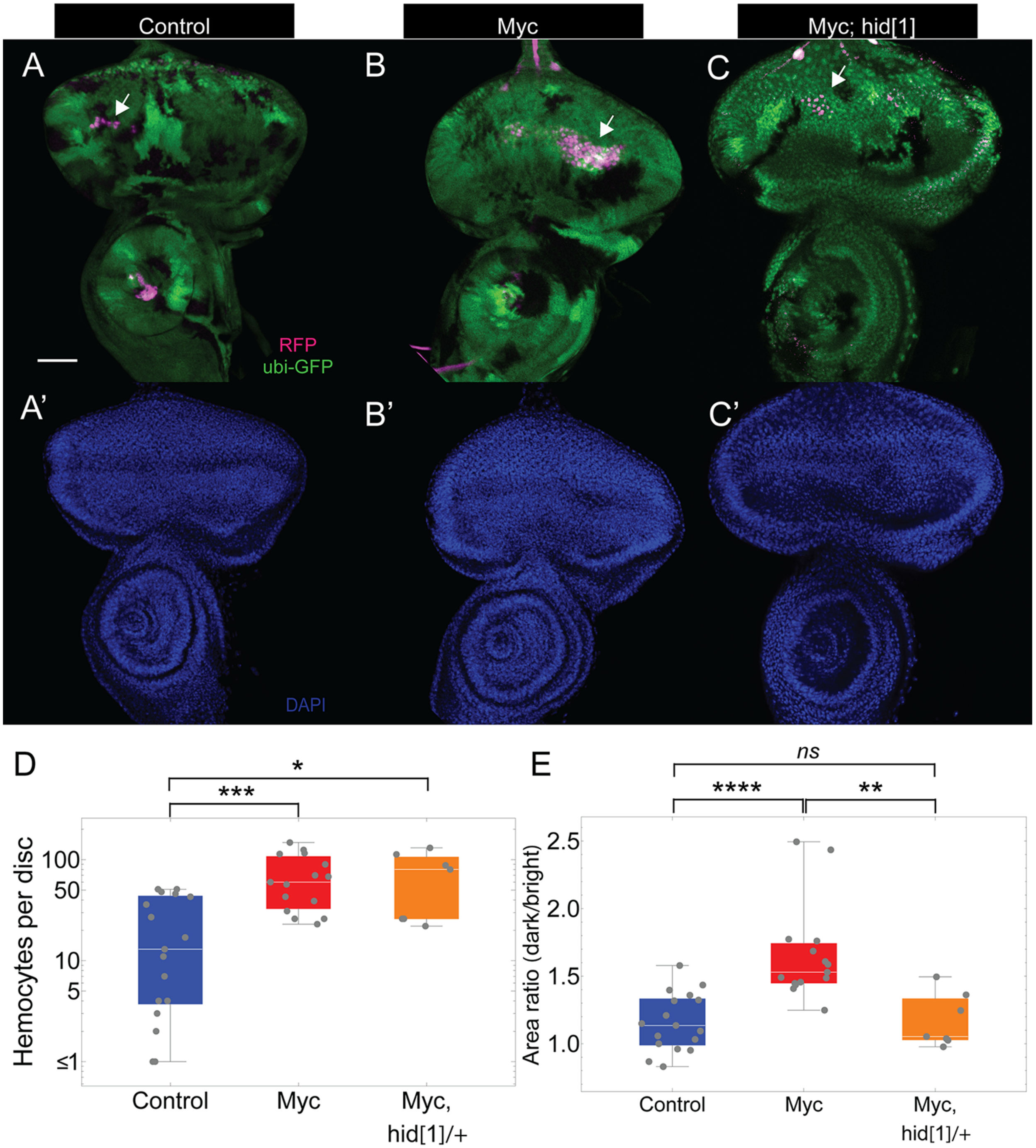

Figure S11, related to Figure 3. Hemocyte recruitment in eye discs when apoptosis is blocked. (**A-C**) Clones were induced in eye discs, and hemocytes detected using *hml-Gal4* driving *UAS-RFP* (indicated by white arrows), 72 h ACI. Control (A) and *dMyc*-overexpressing (B) clones. In (C) clones were generated as in (B), but cell death was blocked using a copy of *hid* allele (hid^1) (C). (**A'-C'**) Discs in (A-C) were stained with DAPI. Scale bar = 50 µm. (**D**) Number of hemocytes per disc for the experiments illustrated in A-C. (**E**) Area ratio between twin clones (dark/bright) in discs of the genotypes in A-C. *=P<0.05; **=P<0.01; ***=P<0.001; ****=P<0.0001, by Mann-Whitney U test. n = 17, 15, and 7 for the experiments illustrated in (A), (B), and (C), respectively. Source data are provided as a Source Data file.

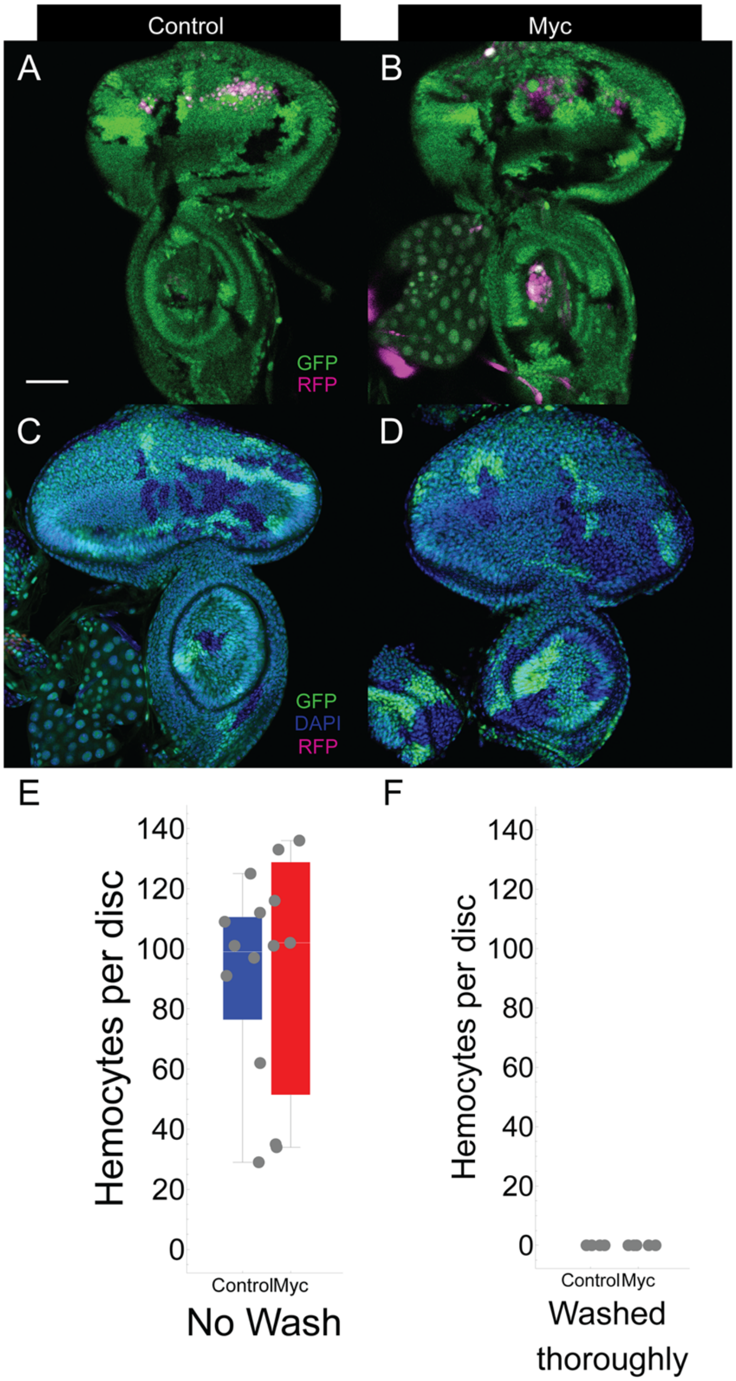

Figure S12, related to Figure 2. Differential effects of washing on hemocytes numbers reveals the influence of cell competition on hemocyte attachment to eye discs. (A-B) Representative eye discs carrying control clones in a wild-type background (A) and *dMyc* clones (B) with hemocytes marked by RFP. Discs were generated and fixed as in (Fig. 3A-B) but imaged without further washes. **(C-D)** Wing discs manipulated as in (A-B), respectively, but fixed for 20 min followed by seven 10-min thorough washes. (**E-F**) Quantification of the number of hemocytes in wing discs of the genotypes in A-B (E), and in C-D (F). n = 8, 7, 4, and 5 for the genotypes in (A), (B), (C), and (D), respectively. Scale bar, 50 μm. Source data are provided as a Source Data file.

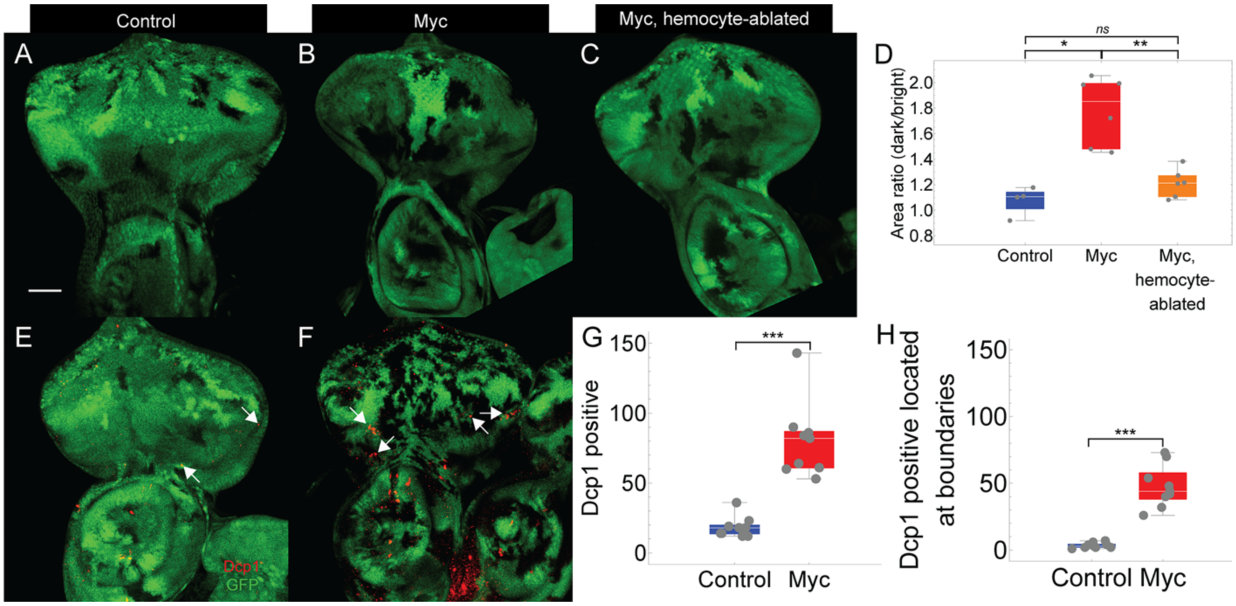

Figure S13, related to Figure 1 and Figure 7. Cell competition is blocked by ablation of hemocytes in eye imaginal discs. (A-C) Representative eye discs carrying twin-spot mosaic clones were induced as in (Fig. 1A-C), 72 h ACI. **(D)** Quantification of the area ratio between twin clones (dark/bright) in eye discs of the genotypes in (A-C). **(E-F)** Eye discs with clones induced as in A-B, respectively, stained with anti-Dcp1 antibodies (red). **(G)** Dcp1-positive cells per disc for the genotypes in E-F. **(H)** The number of Dcp1-positive cells, per disc, located within three cell diameters of clonal boundaries, for the genotypes in E-F. *n* = 4, 6, 6, 9, and 9 for the genotypes in (A), (B), (C), (E), and (F), respectively. Statistical significance was tested using the Mann-Whitney U test (*=P<0.05; **=P<0.01; ***=P<0.001). Scale bar = 50 µm. Source data are provided as a Source Data file.

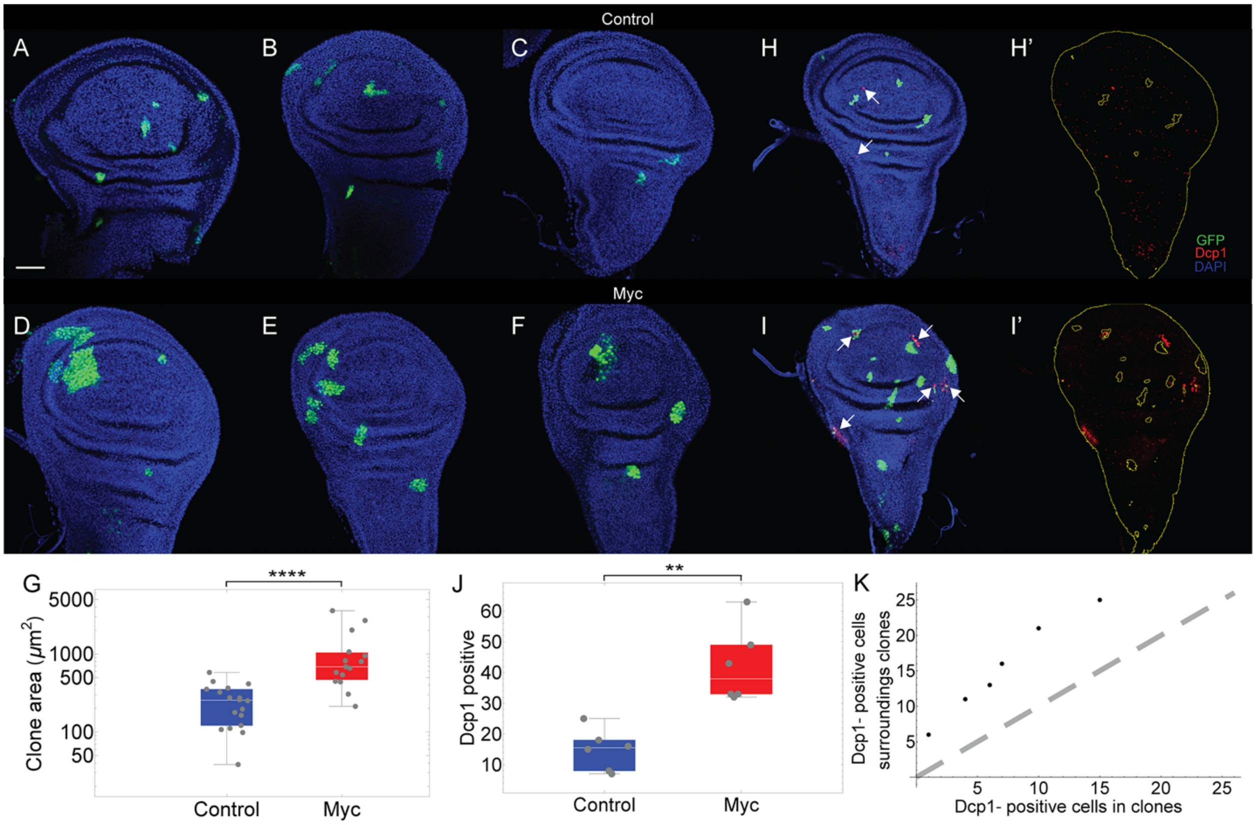

Figure S14, related to Figure 4. Observing cell competition when *dMyc* flip-out clones are generated in a wild-type background. (A-F) Representative wing discs carrying flip-out wild-type clones (A-C) and *dMyc* clones (D-F) stained with DAPI, 48 h ACI. **(G)** Quantification of the size of flip-out clones in wing discs in (A-F) correspondingly. **(H-I')** Representative wing discs with clones induced as in A and D, respectively, stained with anti-Dcp1 antibodies (red). Disc edges and clonal boundaries of discs in H and I were outlined in H' and I', respectively. **(J)** Total Dcp1-positive cells per disc for the genotypes in H-I. *n* = 18, 16, 6, and 6 for the genotypes in (G), and (J), respectively. Statistical significance was tested using the Mann-Whitney U test (**=P<0.01; ****=P<0.0001) and Chi-squared test. Scale bar = 50 µm. **(K)** In each of the six discs with dMyc flp-out clones that were stained for Dcp1, the number of Dcp1-positive cells inside each dMyc clone and the number of Dcp1-positive cells found in an area of equal size immediately surrounding that clone, were measured and totaled; the plot shows that cell death immediately surrounding clones is nearly twice that seen within clones, consistent with preferential killing of wildtype cells. For comparison a line of slope =1 (no preference) is shown. Source data are provided as a Source Data file.

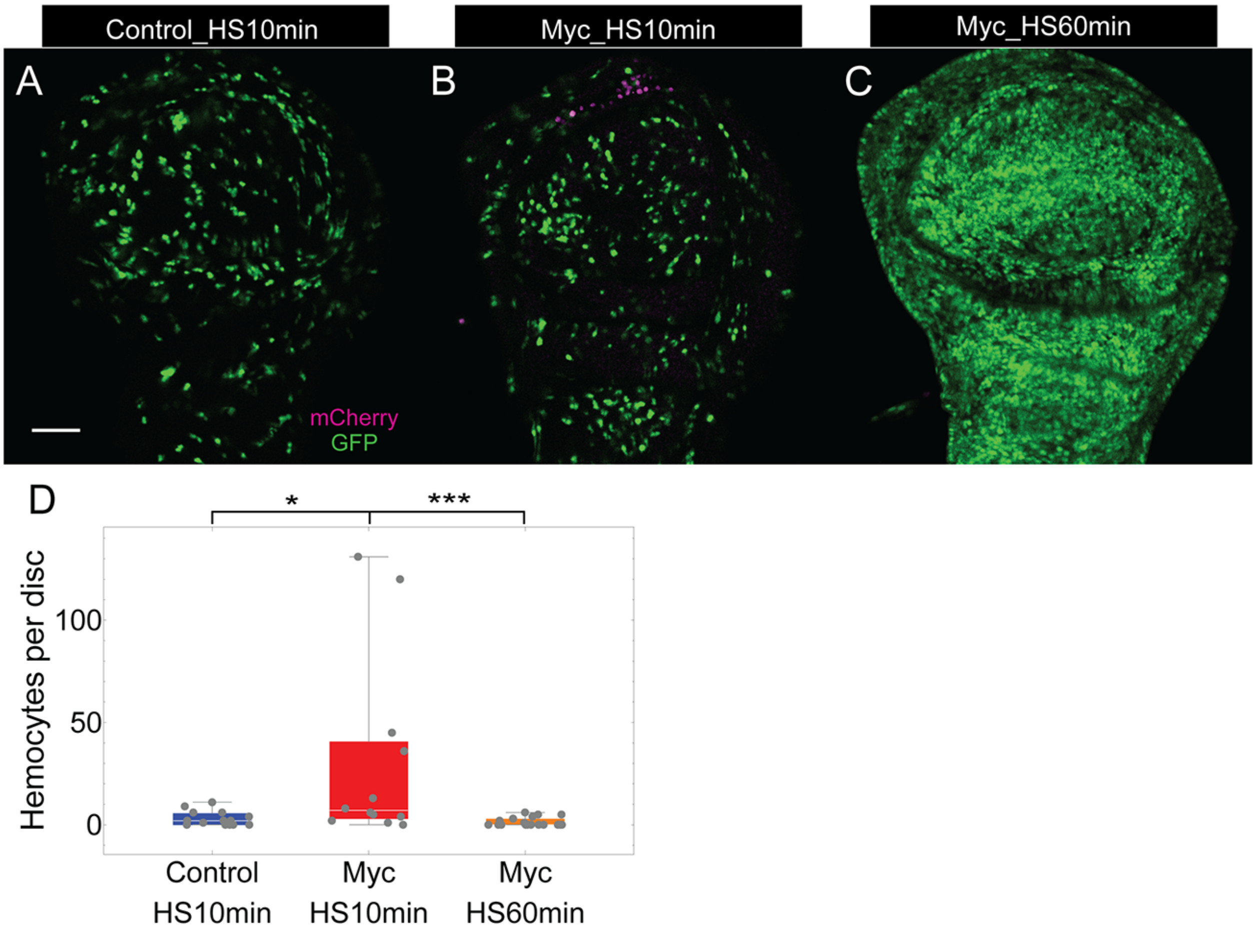

Figure S15, related to Figure 4. Visualizing hemocyte recruitment using an additional fluorescent marker. (**A-C**) Flip-out mosaic clones in wing discs at 14 h ACI. Wild-type (A) and *dMyc*-overexpressing (B,C) clones were created by 10-min (A,B) or 1-hr (C) heat shock. Hemocytes in this case were visualized by *srpHemo-H2A-3xmCherry* (II) instead of *srpHemo-H2A-3xmCherry* (III), as in Fig. 4. (A-C). (**D**) Numbers of hemocytes per wing disc of the genotypes in A-C. *=P<0.05; and ***=P<0.001, by Mann-Whitney U test. n = 15, 12, and 19 for the genotypes in (A), (B), and (C), respectively. Scale bar, 50 μm. Source data are provided as a Source Data file.

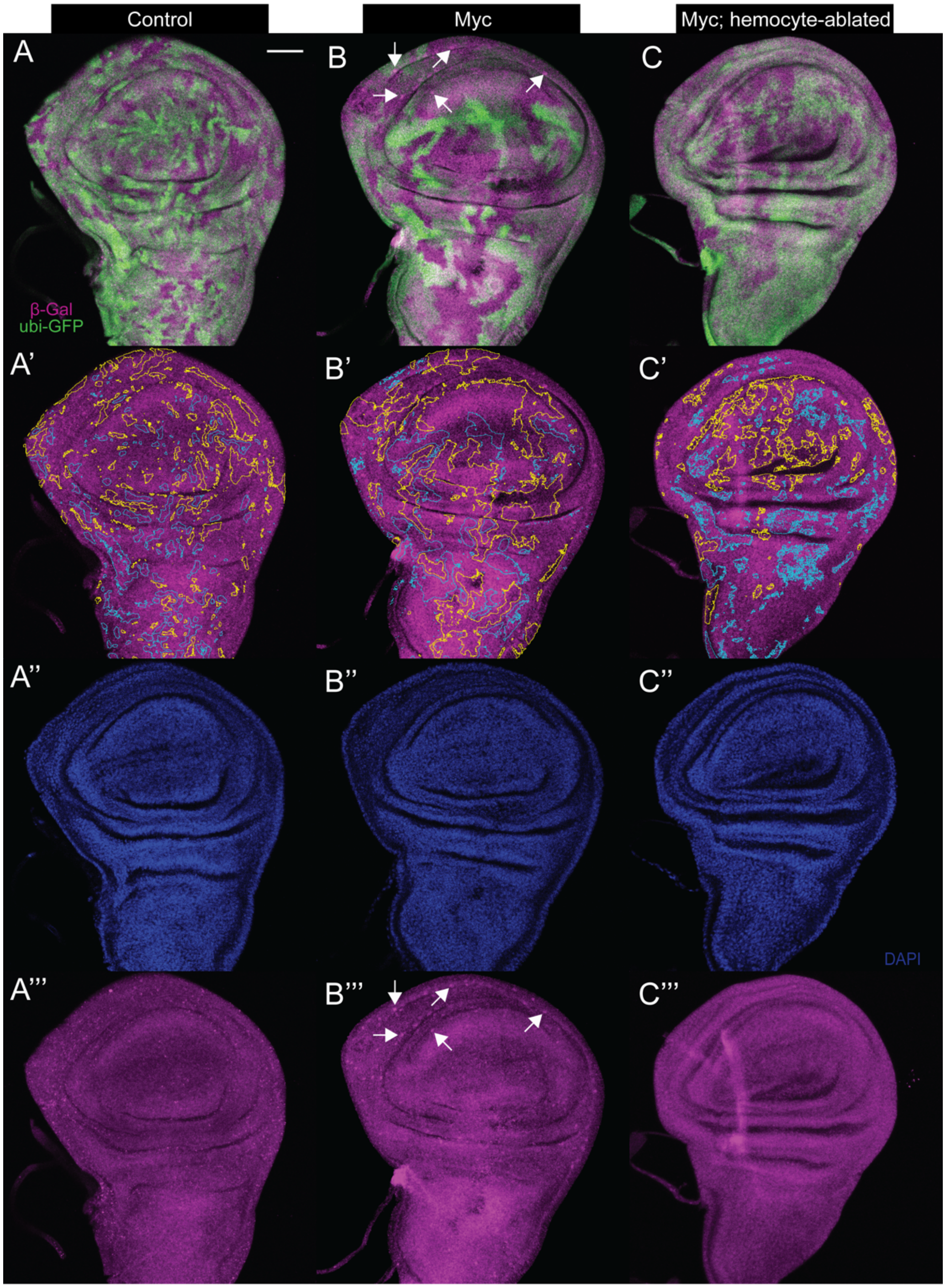

Figure S16. Hemocyte ablation reduces JNK signaling induced by cell competition. (A-C''') Representative wing discs carrying twin-spot mosaic clones as in Fig. 1(A-C) and *puc-lacZ*, a reporter for JNK signaling, 72 h ACI, stained with anti-β-galactosidase (magenta). Areas of elevated JNK signaling are indicated with white arrows (**A'-C'**) On images A-C, clones containing two extra copies of *dMyc* have been outlined by yellow lines and clones containing two copies of GFP (no *dMyc*) have been outlined by blue lines. (**A''-C''**) DAPI staining of discs A-C. (**A'''-C'''**) The maximum intensity projection of anti-β-galactosidase staining of discs A-C. Images are representative of n = 8, 6, and 6 discs for the genotypes in (A), (B), and (C), respectively. Scale bar, 50 μm.

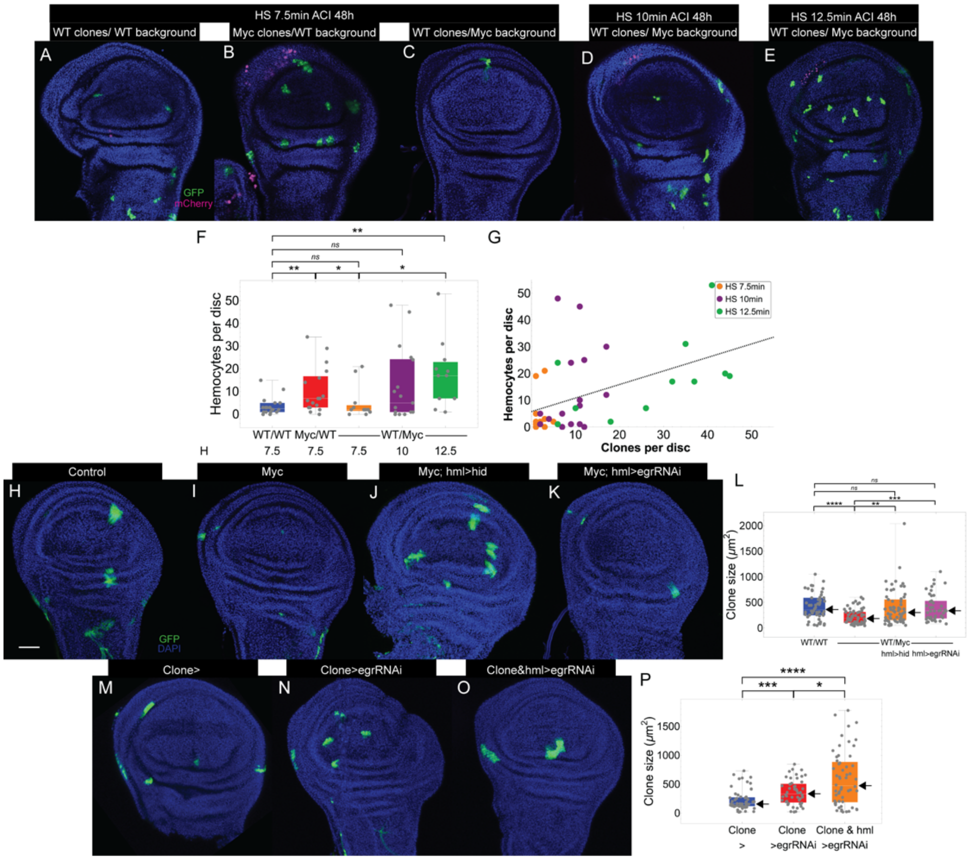

Figure S17, related to Figure 5. The role of hemocytes and hemocyte-derived Egr was tested using an alternate Myc competition system. (**A-E**) In panels A and B, wild-type (A) and *dMyc* (B) clones were generated by 7.5-min heat shock in a wild-type background. In (C-E), wild-type flip-out clones were generated in a *dMyc* background using different heat-shock durations—7.5 (C), 10 (D) or 12.5 (E) min. Hemocytes were visualized using *srpHemo-H2A-3xmCherry* (II) at 48 h ACI. (**F**) Numbers of hemocytes in discs generated as in A-E. *n* = 18, 17, 12, 17, and 11 discs for the genotypes in (A), (B), (C), (D), and (E), respectively. (**G**) For each disc in panel F that contained wild-type flp-out clones in a *dMyc* background, the number of hemocytes was plotted against with the number of clones in the disc. Dashed line show linear fit of the data. (**H-K**) Wild-type flip-out clones were generated in a wild-type background (H) or a *dMyc* background (I) using the LexA system. In (J), clones were induced as in (I), but hemocytes ablated as in Fig. 1C. In (K), clones were created as in (I), but *egr* was knocked-down specifically in hemocytes using RNAi(KK), as in Fig. 5. (**L**) Clone sizes in discs of the genotypes in H-K. *n* = 54, 62, 62, and 42 clones for the genotypes in (H), (I), (J), and (K), respectively. (**M-O**) Wild-type flip-out mosaic clones (M) were induced in a *dMyc* background and observed 48 h ACI. In (N), *egr* was knocked-down in clones, and in (O) eiger was knocked down both in clones and hemocytes, using *egrRNAi*. (**P**) Clone sizes in discs of the genotypes in M-O. *n* = 58, 48, and 56 clones for the genotypes in (M), (N), and (O), respectively. *=$P<0.05$; **=$P<0.01$; ***=$P<0.001$; and ****=$P<0.0001$ (Mann-Whitney U test). Scale bar = 50 µm. *Myc*-induced cell competition assays were as follows: (B) *UAS-Myc* clones in a wild-type background; (C-E) wild-type clones, *tub>Myc* background; (I) wild-type clones, *tub>Myc* background; (J) wild-type clones, *tub>Myc* background with hemocytes ablated; (K) wild-type clones, *tub>Myc* background with *egrRNAi* expressed in hemocytes; (M) wild-type clones, *tub>Myc* background; (N) *egrRNAi* expressed in clones, *tub>Myc* background; (O) *egrRNAi* expressed in both clones and hemocytes, *tub>Myc* background. Source data are provided as a Source Data file.

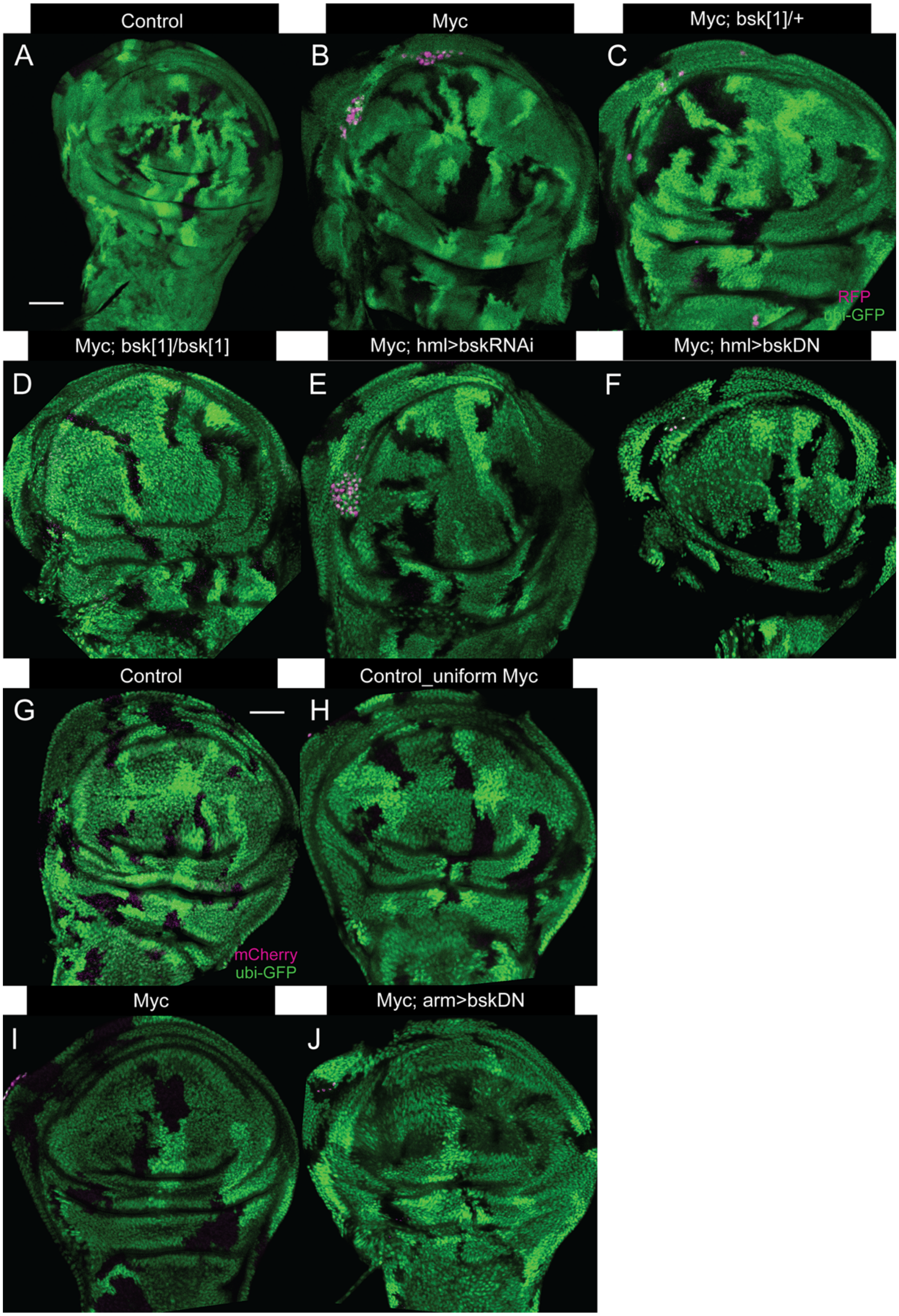

Figure S18, related to Figure 6. Representative wing discs corresponding to genotypes quantified in Figure 6. (**A-F**) Representative wing discs of genotypes in (Fig.6A-B). (**G-J**) Representative wing discs of genotypes in (Fig.6C-D). Scale bar, 50 μm.

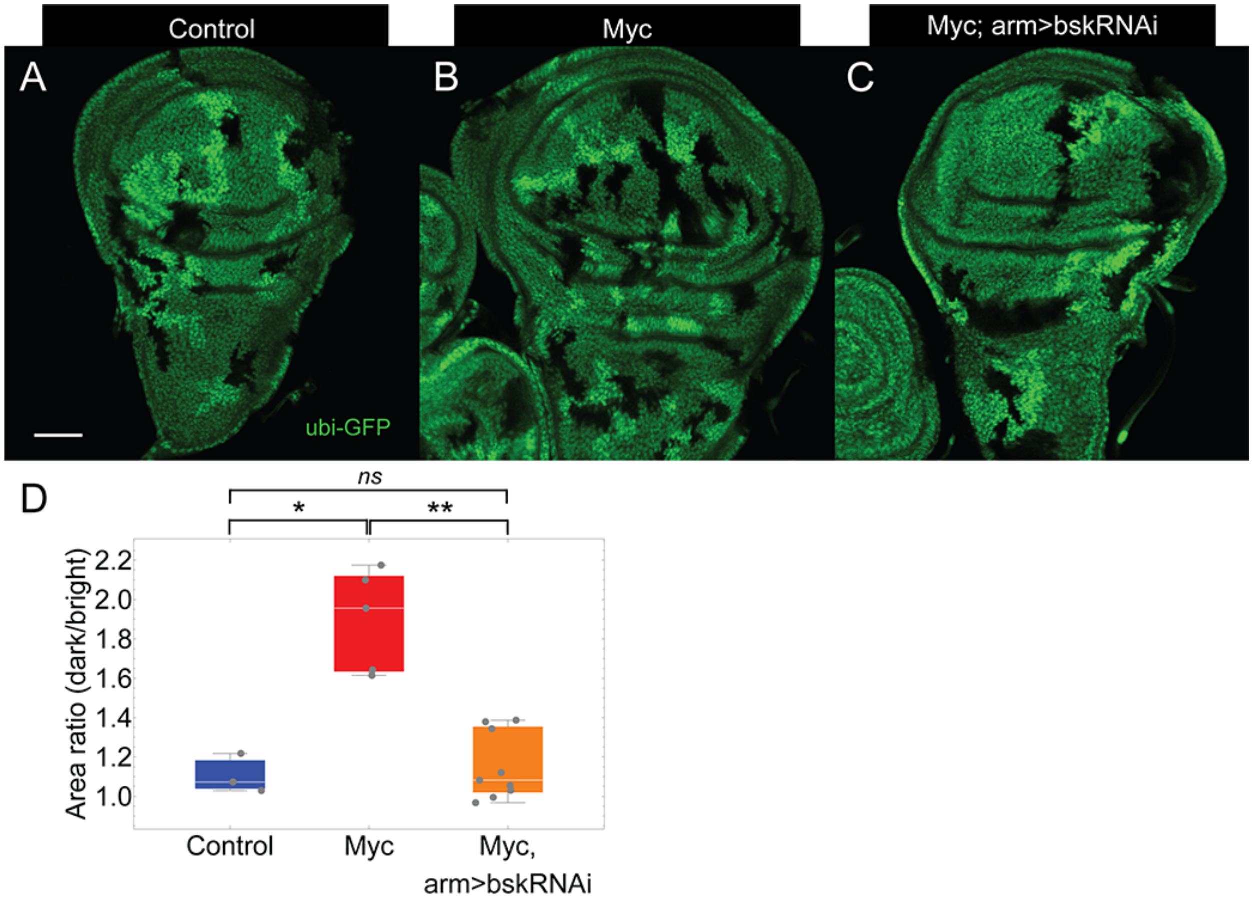

Figure S19, related to Figure 6. Global expression of *bskRNAi* inhibits cell competition. (A-C) Representative wing discs carrying control clones in a wild-type background (A), *dMyc* clones (B) and *dMyc* clones in a background in which *bsk* is knocked down by using *arm-Gal4* to drive *UAS-bskRNAi* (C), 72 h ACI. **(D)** Quantification of the area ratio between twin clones (dark/bright) in wing discs of the genotypes in (A-C). n = 3, 5, and 9 for the genotypes in (A), (B), and (C), respectively. Statistical significance was tested using the Mann-Whitney U test (*=P<0.05; **=P<0.01). Scale bar = 50 μm. Source data are provided as a Source Data file.

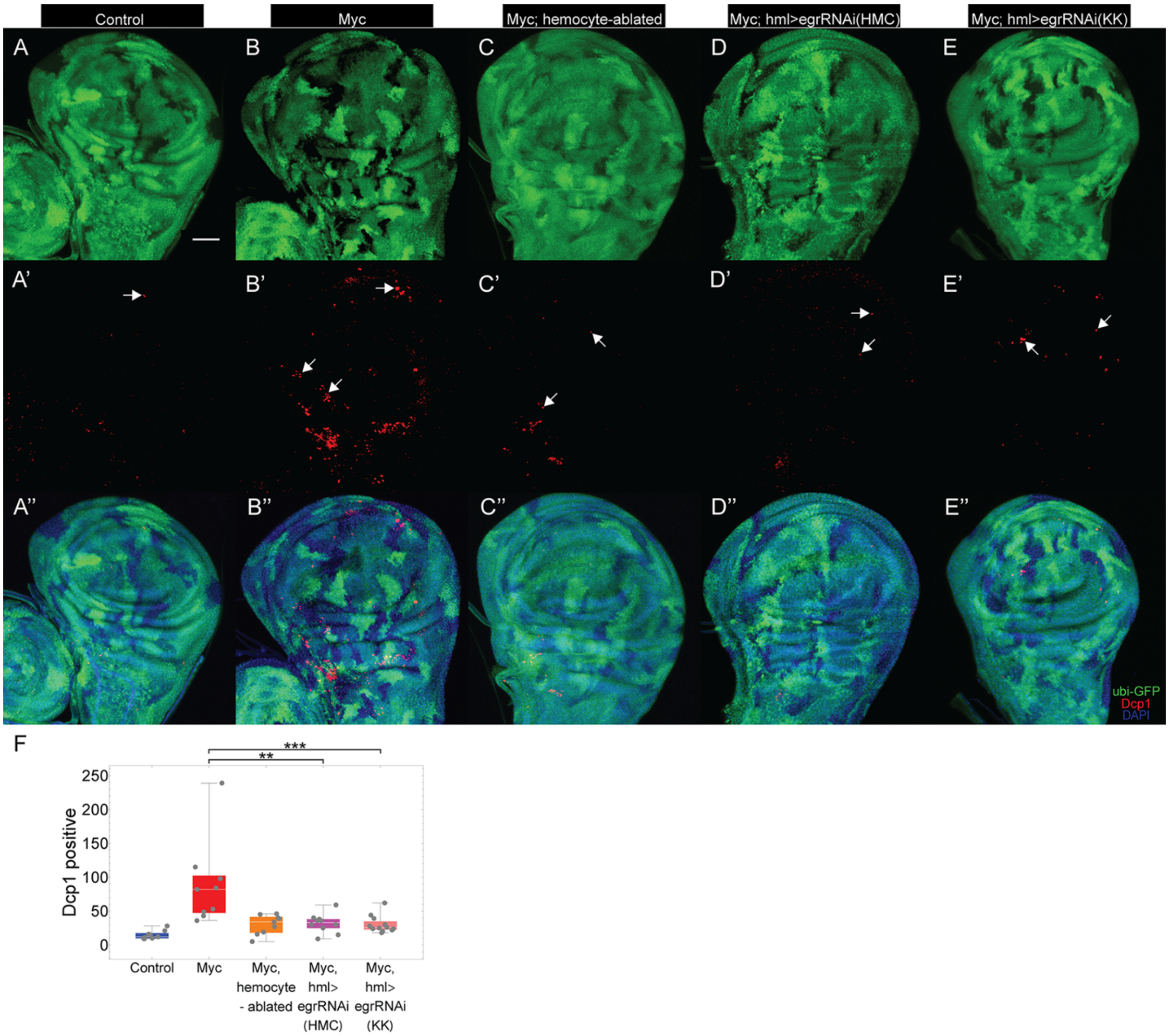

Figure S20, related to Figure 5. Evaluation of cell death in wing discs of genotypes in Figure 5, using anti-Dcp1 immunostaining. (A-E) The maximum projection of wing discs carrying twin-spot mosaic clones induced as in (Fig. 5A-E), 72 h ACI. **(A'-E')** Discs in (A-E) stained with anti-Dcp1 antibody (red), respectively. **(A''-E'')** Discs in (A-E) stained with DAPI (blue), respectively. **(F)** Dcp1-positive cells per disc for the genotypes in A-E. n = 9, 9, 9, 10, and 12 for the genotypes in (A), (B), (C), (D), and (E), respectively. Statistical significance was tested using the Mann-Whitney U test (**=P<0.01; ***=P<0.001). Scale bar = 50 µm. Source data are provided as a Source Data file.

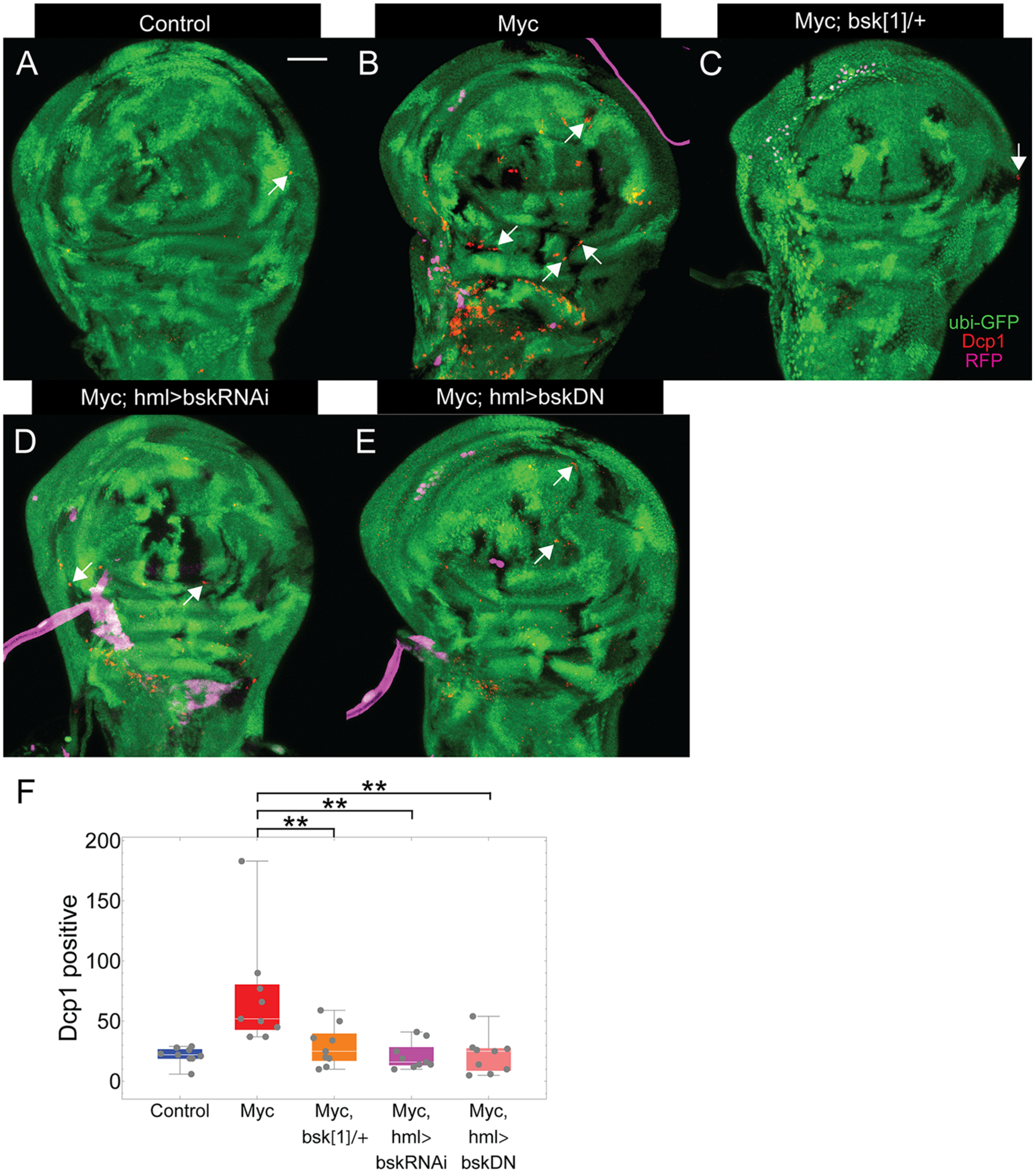

Figure S21, related to Figure 6. Evaluation of cell death in wing discs of genotypes in Figure 6. (A-E) The maximum projection of wing discs carrying twin-spot mosaic clones induced as in (Fig. 6A), 72 h ACI, stained with anti-Dcp1 antibody (red), hemocytes visualized by RFP (magenta), respectively. **(F)** Dcp1-positive cells per disc for the genotypes in A-E. n = 9, 9, 9, 9, and 9 for the genotypes in (A), (B), (C), (D), and (E), respectively. Statistical significance was tested using the Mann-Whitney U test (**=P<0.01). Scale bar = 50 µm. Source data are provided as a Source Data file.

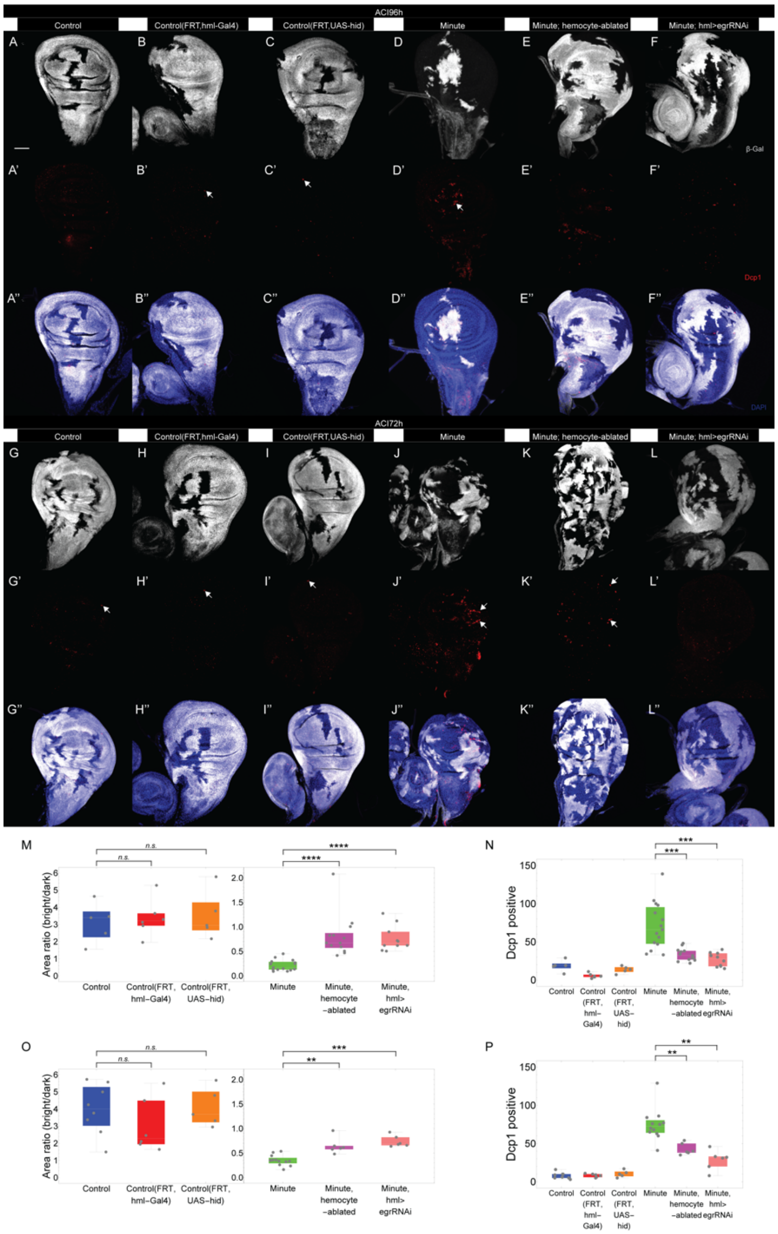

Figure S22. Role of *Eiger* in *Minute*-induced cell competition. In (**A, D and E**) Clones were generated as in Fig. 1, whereas in (**F**) *Minute* twinspot clones were generated, but *egr* was knocked down in hemocytes, using *egrRNAi*. In (**B, C**), control clones were induced in wing discs carrying *hml-Gal4* (B) or *UAS-hid* (C) on the second chromosome. (**A'-F'**) The maximum projection of discs in (A-F) stained with anti-Dcp1 antibody (red), respectively. **(A''-F'')** Discs in (A-F) stained with DAPI (blue), respectively. (**M**) Quantification of the area ratio between clones (bright/dark) in discs of the genotypes in A-F. (**N**) Quantification of the number of Dcp1-positive cells in wing discs of the genotypes in A-F. *n* = 5, 6, 5, 15, 13, and 10 for the genotypes in (A), (B), (C), (D), (E), and (F), respectively. **(G-L'')** Clones were induced as in (A-F''), but wing discs were collected at 72 instead of 96 hours after clone induction. (**O**) Quantification of the area ratio between clones (bright/dark) in discs of the genotypes in G-L. (**P**) Quantification of the number of Dcp1-positive cells in wing discs of the genotypes in G-L. *n* = 8, 6, 5, 12, 6, and 6 for the genotypes in (G), (H), (I), (J), (K), and (L), respectively. **=*P*<0.01; ***=*P*<0.001; ****=P<0.0001 by Mann-Whitney U test. Bar = 50 µm. Source data are provided as a Source Data file.

Table S1. Genotypes used in each Figure

Figure 1: *hsFLP/+; ubi-GFP,FRT40A/FRT40A; +/FRT82B,tub-HA:Myc*$^{w+}$ (A, G); *hsFLP/+;; FRT82B,ubi-GFP/FRT82B,tub-HA:Myc*$^{w+}$ (B, G); *hsFLP/+; UAS-hid/hml-Gal4; FRT82B,ubi-GFP/FRT82B,tub-HA:Myc*$^{w+}$ (C, G); *hsFLP/+; FRT40A/arm-lacZ,FRT40A* (D, H); *hsFLP/+; FRT40A/arm-lacZ,M(2)Z,FRT40A* (E, H); *hsFLP/+; FRT40A,UAS-hid/ arm-lacZ,M(2)Z,FRT40A; hml-Gal4/+* (F, H).

Figure 2: *UAS-RFP/hml-Gal4* (A, A', D); *hsFLP/+; ubi-GFP,FRT40A/FRT40A; UAS-RFP/hml-Gal4* (B, B', D); *hsFLP/+;UAS-RFP/hml-Gal4; FRT82B,ubi-GFP/FRT82B,tub-HA:Myc*$^{w+}$ (C, C', D, E, G-I); *hsFLP/+; UAS-RFP/hml-Gal4; FRT82B,ubi-GFP/FRT82B* (F, F', H, I).

Figure 3: *hsFLP/+; UAS-RFP/hml-Gal4; FRT82B,ubi-GFP/FRT82B* (A, A', G, H); *hsFLP/+;UAS-RFP/hml-Gal4; FRT82B,ubi-GFP/FRT82B,tub-HA:Myc*$^{w+}$ (B, B', G, H); *hsFLP/+;UAS-RFP/hml-Gal4; FRT82B,ubi-GFP,hid*^{1}*/FRT82B,tub-HA:Myc*$^{w+}$ (C, C', G, H); *hsFLP/+; +/srpHemo-H2A.3XmCherry; FRT82B,ubi-GFP/FRT82B* (D, D', I, J); *hsFLP/+; +/srpHemo-H2A.3XmCherry; FRT82B,ubi-GFP/FRT82B,tub-HA:Myc*$^{w+}$ (E, E', I, J); *hsFLP/+; UAS-p35/arm-Gal4,srpHemo-H2A.3XmCherry; FRT82B,ubi-GFP/FRT82B,tub-HA:Myc*$^{w+}$ (F, F', I, J).

Figure 4: *hsFLP/+;; Act>y>Gal4, UAS-GFP/srpHemo-H2A.3XmCherry* (A, G); *hsFLP/+; UAS-Myc/+; Act>y>Gal4, UAS-GFP/srpHemo-H2A.3XmCherry* (B, C, G); *hsFLP/+; UAS-RFP/hml-Gal4; FRT82B,ubi-GFP/FRT82B* (D, D', H); *hsFLP/+;UAS-RFP/hml-Gal4; FRT82B,ubi-GFP/FRT82B,tub-HA:Myc*$^{w+}$ (E, E', H); *hsFLP/+;UAS-RFP/hml-Gal4; FRT82B, tub-HA:Myc*$^{w+}$*/FRT82B,tub-HA:Myc*$^{w+}$ (F, F', H).

Figure 5: *hsFLP/+; ubi-GFP,FRT40A/FRT40A; +/FRT82B,tub-HA:Myc*$^{w+}$ (A, F); *hsFLP/+;; FRT82B,ubi-GFP/FRT82B,tub-HA:Myc*$^{w+}$ (B, F); *hsFLP/+; UAS-hid/hml-Gal4; FRT82B,ubi-GFP/FRT82B,tub-HA:Myc*$^{w+}$ (C, F); *hsFLP/+; UAS-egrRNAi(HMC)/hml-Gal4; FRT82B,ubi-GFP/FRT82B,tub-HA:Myc*$^{w+}$ (D, F); *hsFLP/+; UAS-egrRNAi(KK)/hml-Gal4; FRT82B,ubi-GFP/FRT82B,tub-HA:Myc*$^{w+}$ (E, F); *hsFLP/+; UAS-RFP/hml-Gal4; FRT82B,ubi-GFP/FRT82B* (G, K, L); *hsFLP/+;UAS-RFP/hml-Gal4; FRT82B,ubi-GFP/FRT82B,tub-HA:Myc*$^{w+}$ (H, K, L); *hsFLP/+;UAS-egrRNAi(HMC)/hml-Gal4,UAS-RFP; FRT82B,ubi-GFP/FRT82B,tub-HA:Myc*$^{w+}$ (I, K, L); *hsFLP/+;UAS-egrRNAi(KK)/hml-Gal4,UAS-RFP; FRT82B,ubi-GFP/FRT82B,tub-HA:Myc*$^{w+}$ (J, K, L).

Figure 6: *hsFLP/+; UAS-RFP/hml-Gal4; FRT82B,ubi-GFP/FRT82B* (A, B); *hsFLP/+; UAS-RFP/hml-Gal4; FRT82B,ubi-GFP/FRT82B,tub-HA:Myc*$^{w+}$ (A, B); *hsFLP/+; UAS-RFP,bsk*^{1}*/hml-Gal4; FRT82B,ubi-GFP/FRT82B,tub-HA:Myc*$^{w+}$ (A, B); *hsFLP/+; UAS-RFP,bsk*^{1}*/hml-Gal4,bsk*^{1}*; FRT82B,ubi-GFP/FRT82B,tub-HA:Myc*$^{w+}$ (A, B); *hsFLP/+; UAS-bskRNAi/hml-Gal4,UAS-RFP; FRT82B,ubi-GFP/FRT82B,tub-HA:Myc*$^{w+}$ (A, B); *hsFLP/UAS-bsk*^{DN}*; UAS-RFP/hml-Gal4; FRT82B,ubi-GFP/FRT82B,tub-HA:Myc*$^{w+}$ (A, B); *hsFLP/+; +/srpHemo-H2A.3XmCherry; FRT82B,ubi-GFP/FRT82B* (C, D); *hsFLP/+; FRT40A,ubi-GFP/FRT40A; srpHemo-H2A.3XmCherry/FRT82B,tub-HA:Myc*$^{w+}$ (C, D); *hsFLP/+; +/srpHemo-H2A.3XmCherry; FRT82B,ubi-GFP/FRT82B,tub-HA:Myc*$^{w+}$ (C, D); *hsFLP/UAS-bsk*^{DN}*; arm-Gal4/srpHemo-H2A.3XmCherry; FRT82B,ubi-GFP/FRT82B,tub-HA:Myc*$^{w+}$ (C, D).

Figure 7: *hsFLP/+; ubi-GFP,FRT40A/FRT40A; +/FRT82B,tub-HA:Myc*$^{w+}$ (B, B', B'', E, E', E'', H, I); *hsFLP/+;; FRT82B,ubi-GFP/FRT82B,tub-HA:Myc*$^{w+}$ (A, A', A'', C, C', C'', F, F', F'', H, I); *hsFLP/+; UAS-hid/hml-Gal4; FRT82B,ubi-GFP/FRT82B,tub-HA:Myc*$^{w+}$ (D, D', D'', G, G', G'', H, I).

Figure S2: *hsFLP/+;; FRT82B,ubi-GFP/FRT82B,tub-HA:Myc*$^{w+}$ (A-E); *hsFLP/+; FRT40A/arm-lacZ,M(2)Z,FRT40A* (F-H).

Figure S3: *srpHemo-H2A.3XmCherry* (A, C, E, F); *UAS-hid/hml-Gal4; srpHemo-H2A.3XmCherry/+* (B, D, E, F).

Figure S4: *wild-type* (A, A', A'', A''', A'''', E); *UAS-hid/hml-Gal4* (B, B',B'', B''', B'''', E); *arm-lacZ,M(2)Z,FRT40A/+* (C, C', C'', C''', C'''', F); *arm-lacZ,M(2)Z,FRT40A/UAS-hid; +/hml-Gal4* (D, D', D'', D''', D'''', F).

Figure S5: *hsFLP/+; FRT40A/arm-lacZ,FRT40A* (A, D, E); *hsFLP/+; FRT40A/arm-lacZ,M(2)Z,FRT40A* (B, D, E); *hsFLP/+; FRT40A,UAS-hid/ arm-lacZ,M(2)Z,FRT40A; hml-Gal4/+* (C-E).

Figure S6: *hsFLP/+; ubi-GFP,FRT40A/FRT40A; UAS-RFP/hml-Gal4* (A-A'', C-C'', E-E''); *hsFLP/+;UAS-RFP/hml-Gal4; FRT82B,ubi-GFP/FRT82B,tub-HA:Myc*$^{w+}$ (B-B'', D-D'', F-F'').

Figure S7: *hsFLP/+; +/srpHemo-3XmCherry; FRT82B,ubi-GFP/FRT82B* (A, D); *hsFLP/+; FRT40A,ubi-GFP/FRT40A; srpHemo-3XmCherry/FRT82B,tub-HA:Myc*$^{w+}$ (B, D); *hsFLP/+; +/srpHemo-3XmCherry; FRT82B,ubi-GFP/FRT82B,tub-HA:Myc*$^{w+}$ (C, D);

Figure S8: *hsFLP/+; +/srpHemo-H2A.3XmCherry; Act>y>Gal4, UAS-GFP/+* (A, D, G, J); *hsFLP/+; UAS-Myc/srpHemo-H2A.3XmCherry; Act>y>Gal4, UAS-GFP/+* (B, C, E, F, H-J); *hsFLP/+;; Act>y>Gal4, UAS-GFP/srpHemo-H2A.3XmCherry* (K); *hsFLP/+; UAS-Myc/+; Act>y>Gal4, UAS-GFP/srpHemo-H2A.3XmCherry* (K).

Figure S9: *hsFLP/+; FRT40A/arm-lacZ,FRT40A; srpHemo-H2A.3XmCherry/*+ (A, A', C); *hsFLP/+; FRT40A/arm-lacZ,M(2)Z,FRT40A; srpHemo-H2A.3XmCherry/*+ (B, B', C).

Figure S10: *hsFLP/+; UAS-RFP/hml-Gal4; FRT82B,ubi-GFP/FRT82B* (A, C, E-J, Q, R, R', T-V); *hsFLP/+;UAS-RFP/hml-Gal4; FRT82B,ubi-GFP/FRT82B,tub-HA:Myc*$^{w+}$ (B, D, K-Q, S-V).

Figure S11: *hsFLP/+; UAS-RFP/hml-Gal4; FRT82B,ubi-GFP/FRT82B* (A, A', D, E); *hsFLP/+;UAS-RFP/hml-Gal4; FRT82B,ubi-GFP/FRT82B,tub-HA:Myc*$^{w+}$ (B, B', D, E); *hsFLP/+;UAS-RFP/hml-Gal4; FRT82B,ubi-GFP,hid*^{1}*/FRT82B,tub-HA:Myc*$^{w+}$ (C-E).

Figure S12: *hsFLP/+; UAS-RFP/hml-Gal4; FRT82B,ubi-GFP/FRT82B* (A, C, E, F); *hsFLP/+;UAS-RFP/hml-Gal4; FRT82B,ubi-GFP/FRT82B,tub-HA:Myc*$^{w+}$ (B, D, E, F).

Figure S13: *hsFLP/+; ubi-GFP,FRT40A/FRT40A; +/FRT82B,tub-HA:Myc*$^{w+}$ (A, D, E, G, H); *hsFLP/+;; FRT82B,ubi-GFP/FRT82B,tub-HA:Myc*$^{w+}$ (B, D, F, G, H); *hsFLP/+; UAS-hid/hml-Gal4; FRT82B,ubi-GFP/FRT82B,tub-HA:Myc*$^{w+}$ (C, D);

Figure S14: *hsFLP/+;; Act>y>Gal4, UAS-GFP/*+ (A-C, G-H', J); *hsFLP/+; UAS-Myc/+; Act>y>Gal4, UAS-GFP/*+ (D-G, I-K).

Figure S15: *hsFLP/+; +/srpHemo-H2A.3XmCherry; Act>y>Gal4, UAS-GFP/*+ (A, D); *hsFLP/+; UAS-Myc/srpHemo-H2A.3XmCherry; Act>y>Gal4, UAS-GFP/*+ (B, C, D).

Figure S16: *hsFLP/+;; puc-lacZ,FRT82B,ubi-GFP/FRT82B* (A, A', A'', A'''); *hsFLP/+;; puc-lacZ,FRT82B,ubi-GFP/FRT82B,tub-HA:Myc*$^{w+}$ (B, B', B'', B'''); *hsFLP/+; UAS-hid/hml-Gal4; puc-lacZ,FRT82B,ubi-GFP/FRT82B,tub-HA:Myc*$^{w+}$ (C, C', C'', C''').

Figure S17: *hsFLP/+; +/srpHemo-H2A.3XmCherry; Act>y>Gal4, UAS-GFP/*+ (A, F); *hsFLP/+; UAS-Myc/srpHemo-H2A.3XmCherry; Act>y>Gal4, UAS-GFP/*+ (B, F); *hsFLP,tub>Myc>Gal4/+; UAS-GFP/srpHemo-H2A.3XmCherry* (C-G); *hsFLP;; tub>CD2>LexA/LexOP-GFP* (H, L); *hsFLP;; tub>Myc>LexA/LexOP-GFP* (I, L); *hsFLP;UAS-hid/hml-Gal4; tub>Myc>LexA/LexOP-GFP* (J, L); *hsFLP;UAS-egrRNAi(KK)/hml-Gal4; tub>Myc>LexA/LexOP-GFP* (K, L); *hsFLP,tub>Myc>Gal4/+; UAS-GFP/*+ (M, P); *hsFLP,tub>Myc>Gal4/+; UAS-GFP/+; +/UAS-egrRNAi* (N, P); *hsFLP,tub>Myc>Gal4/+; UAS-GFP/hml-Gal4; +/UAS-egrRNAi* (O, P);

Figure S18: *hsFLP/+; UAS-RFP/hml-Gal4; FRT82B,ubi-GFP/FRT82B* (A); *hsFLP/+; UAS-RFP/hml-Gal4; FRT82B,ubi-GFP/FRT82B,tub-HA:Myc*$^{w+}$ (B); *hsFLP/+; UAS-RFP,bsk*^{1}*/hml-Gal4; FRT82B,ubi-GFP/FRT82B,tub-HA:Myc*$^{w+}$ (C); *hsFLP/+; UAS-RFP,bsk*^{1}*/hml-Gal4,bsk*^{1}*; FRT82B,ubi-GFP/FRT82B,tub-HA:Myc*$^{w+}$ (D); *hsFLP/+; UAS-bskRNAi/hml-Gal4,UAS-RFP; FRT82B,ubi-GFP/FRT82B,tub-HA:Myc*$^{w+}$ (E); *hsFLP/UAS-bsk*^{DN}*; UAS-RFP/hml-Gal4; FRT82B,ubi-GFP/FRT82B,tub-HA:Myc*$^{w+}$ (F); *hsFLP/+; +/srpHemo-H2A.3XmCherry; FRT82B,ubi-GFP/FRT82B* (G); *hsFLP/+; FRT40A,ubi-GFP/FRT40A; srpHemo-H2A.3XmCherry/FRT82B,tub-HA:Myc*$^{w+}$ (H); *hsFLP/+; +/srpHemo-H2A.3XmCherry; FRT82B,ubi-GFP/FRT82B,tub-HA:Myc*$^{w+}$ (I); *hsFLP/UAS-bsk*^{DN}*; arm-Gal4/srpHemo-H2A.3XmCherry; FRT82B,ubi-GFP/FRT82B,tub-HA:Myc*$^{w+}$ (J).

Figure S19: *hsFLP/+;; FRT82B,ubi-GFP/FRT82B* (A, D); *hsFLP/+;; FRT82B,ubi-GFP/FRT82B,tub-HA:Myc*$^{w+}$ (B, D); *hsFLP/+; UAS-bskRNAi/arm-Gal4; FRT82B,ubi-GFP/FRT82B,tub-HA:Myc*$^{w+}$ (C, D).

Figure S20: *hsFLP/+; ubi-GFP,FRT40A/FRT40A; +/FRT82B,tub-HA:Myc*$^{w+}$ (A, A', A'', F); *hsFLP/+;; FRT82B,ubi-GFP/FRT82B,tub-HA:Myc*$^{w+}$ (B, B', B'', F); *hsFLP/+; UAS-hid/hml-Gal4; FRT82B,ubi-GFP/FRT82B,tub-HA:Myc*$^{w+}$ (C, C', C'', F); *hsFLP/+; UAS-egrRNAi(HMC)/hml-Gal4; FRT82B,ubi-GFP/FRT82B,tub-HA:Myc*$^{w+}$ (D, D', D'', F); *hsFLP/+; UAS-egrRNAi(KK)/hml-Gal4; FRT82B,ubi-GFP/FRT82B,tub-HA:Myc*$^{w+}$ (E, E', E'', F)

Figure S21: *hsFLP/+; UAS-RFP/hml-Gal4; FRT82B,ubi-GFP/FRT82B* (A, F); *hsFLP/+; UAS-RFP/hml-Gal4; FRT82B,ubi-GFP/FRT82B,tub-HA:Myc*$^{w+}$ (B, F); *hsFLP/+; UAS-RFP,bsk*^{1}*/hml-Gal4; FRT82B,ubi-GFP/FRT82B,tub-HA:Myc*$^{w+}$ (C, F); *hsFLP/+; UAS-bskRNAi/hml-Gal4,UAS-RFP; FRT82B,ubi-GFP/FRT82B,tub-HA:Myc*$^{w+}$ (D, F); *hsFLP/UAS-bsk*^{DN}*; UAS-RFP/hml-Gal4; FRT82B,ubi-GFP/FRT82B,tub-HA:Myc*$^{w+}$ (E, F).

Figure S22: *hsFLP/+; FRT40A/arm-lacZ,FRT40A* (A, A', A'', G, G', G'', M-P); *hsFLP/+; FRT40A,hml-Gal4/arm-lacZ,FRT40A* (B, B', B'', H, H', H'', M-P); *hsFLP/+; FRT40A,UAS-hid/arm-lacZ,FRT40A* (C, C', C'', I, I', I'', M-P); *hsFLP/+; FRT40A/arm-lacZ,M(2)Z,FRT40A* (D, D', D'', J, J', J'', M-P); *hsFLP/+; FRT40A,UAS-hid/arm-lacZ,M(2)Z,FRT40A; hml-Gal4/*+ (E, E', E'' , K, K', K'', M-P); *hsFLP/+; FRT40A,hml-Gal4/arm-lacZ,M(2)Z,FRT40A; UAS-egrRNAi/*+ (F, F', F'', L,

L’, L’’, M-P).

Table S2: P-values and statistical tests used in each figure

Figure	Panel	Comparsion	P-value	Statistical test
1	G	Control vs Myc	$6.45x10^{-7}$	Mann-Whitney U test
		Myc vs Myc, hemocyte-ablated	$1.35x10^{-6}$	Mann-Whitney U test
	H	Control vs Minute	$7.08x10^{-6}$	Mann-Whitney U test
		Minute vs Minute, hemocyte-ablated	$1.41x10^{-5}$	Mann-Whitney U test
2	D	Control vs Myc 16 h	$8.95x10^{-4}$	Mann-Whitney U test
		Control vs Myc 24 h	$1.23x10^{-2}$	Mann-Whitney U test
		Control vs Myc 48 h	$4.27x10^{-3}$	Mann-Whitney U test
		Control vs Myc 72 h	$1.86x10^{-2}$	Mann-Whitney U test
	H	Control vs Myc	$3.44x10^{-3}$	Mann-Whitney U test
	I	Control vs Myc	$3.42x10^{-4}$	
3	G	Control vs Myc	$6.65x10^{-3}$	Mann-Whitney U test
		Control vs Myc, hid[1]/+	$1.78x10^{-3}$	Mann-Whitney U test
	H	Control vs Myc	$1.25x10^{-6}$	Mann-Whitney U test
		Myc vs Myc, hid[1]/+	$1.65x10^{-5}$	Mann-Whitney U test
	I	Control vs Myc	$1.23x10^{-3}$	Mann-Whitney U test
		Control vs Myc, arm>p35	$2.91x10^{-3}$	Mann-Whitney U test
	J	Control vs Myc	$3.51x10^{-7}$	Mann-Whitney U test
		Myc vs Myc, arm>p35	$1.41x10^{-6}$	Mann-Whitney U test
4	G	Control HS 10min vs Myc HS 10min	$8.14x10^{-3}$	Mann-Whitney U test
		Myc HS 10min vs Myc HS 60min	$3.19x10^{-3}$	Mann-Whitney U test
	H	Myc vs Uniform 2x Myc	$1.49x10^{-2}$	Mann-Whitney U test
5	F	Control vs Myc	$1.08x10^{-4}$	Mann-Whitney U test

		Myc vs Myc, hemocyte-ablated	1.90×10^{-4}	Mann-Whitney U test
		Myc vs Myc, hml>egrRNAi(HMC)	5.92×10^{-3}	Mann-Whitney U test
		Myc vs Myc, hml>egrRNAi(KK)	1.94×10^{-3}	Mann-Whitney U test
	K	Control vs Myc	6.66×10^{-3}	Mann-Whitney U test
		Control vs Myc, hml>egrRNAi(HMC)	2.44×10^{-1}	Mann-Whitney U test
		Control vs Myc, hml>egrRNAi(KK)	2.55×10^{-1}	Mann-Whitney U test
		Myc vs Myc, hml>egrRNAi(HMC)	2.69×10^{-2}	Mann-Whitney U test
		Myc vs Myc, hml>egrRNAi(KK)	4.85×10^{-2}	Mann-Whitney U test
	L	Control vs Myc	2.96×10^{-6}	Mann-Whitney U test
		Control vs Myc, hml>egrRNAi(HMC)	1.44×10^{-1}	Mann-Whitney U test
		Control vs Myc, hml>egrRNAi(KK)	5.12×10^{-2}	Mann-Whitney U test
		Myc vs Myc, hml>egrRNAi(HMC)	4.67×10^{-5}	Mann-Whitney U test
		Myc vs Myc, hml>egrRNAi(KK)	1.41×10^{-4}	Mann-Whitney U test
6	A	Control vs Myc, bsk[1]/bsk[1]	1.01×10^{-2}	Mann-Whitney U test
		Myc vs Myc, bsk[1]/bsk[1]	1.00×10^{0}	Mann-Whitney U test
	B	Myc vs Myc, bsk[1]/+	3.01×10^{-5}	Mann-Whitney U test
		Myc vs Myc, bsk[1]/bsk[1]	2.15×10^{-4}	Mann-Whitney U test
		Myc vs Myc, hml>bskRNAi	1.57×10^{-4}	Mann-Whitney U test
		Myc vs Myc, hml>bskDN	1.36×10^{-4}	Mann-Whitney U test
	C	Control vs Myc	5.83×10^{-4}	Mann-Whitney U test

		Control vs Myc, arm>bskDN	1.13×10^{-2}	Mann-Whitney U test
		Control, uniform Myc vs Myc	5.83×10^{-4}	Mann-Whitney U test
		Control, uniform Myc vs Myc, arm>bskDN	3.30×10^{-2}	Mann-Whitney U test
		Myc vs Myc, arm>bskDN	1.00×10^{-1}	Mann-Whitney U test
	D	Control vs Myc	1.57×10^{-4}	Mann-Whitney U test
		Control, uniform Myc vs Myc	1.57×10^{-4}	Mann-Whitney U test
		Myc vs Myc, arm>bskDN	5.30×10^{-4}	Mann-Whitney U test
7	H	Myc, No heat-shock vs Myc	8.88×10^{-5}	Mann-Whitney U test
		Control vs Myc	2.48×10^{-5}	Mann-Whitney U test
		Myc vs Myc, hemocyte-ablated	2.09×10^{-4}	Mann-Whitney U test
	I	Control vs Myc	1.24×10^{-4}	Mann-Whitney U test
		Control vs Myc, hemocyte-ablated	6.20×10^{-2}	Mann-Whitney U test
		Myc vs Myc, hemocyte-ablated	3.45×10^{-4}	Mann-Whitney U test
S3	F	Wild-type vs Hemocyte-ablated	4.82×10^{-5}	Mann-Whitney U test
S5	E	Control vs Minute	7.08×10^{-6}	Mann-Whitney U test
		Minute vs Minute, hemocyte-ablated	2.09×10^{-4}	Mann-Whitney U test
S7	D	Control vs Myc	3.86×10^{-4}	Mann-Whitney U test
		Control, uniform Myc vs Myc	1.27×10^{-2}	Mann-Whitney U test
S9	C	Control vs Minute	3.67×10^{-2}	Mann-Whitney U test
S10	Q	Control vs Myc 4	1.50×10^{-3}	Mann-Whitney U test
		Control vs Myc 5	3.75×10^{-3}	Mann-Whitney U test
	U	Control vs Myc	1.40×10^{-11}	Mann-Whitney U test

S11	D	Control vs Myc	5.89×10^{-4}	Mann-Whitney U test
		Control vs Myc, hid[1]/+	1.45×10^{-2}	Mann-Whitney U test
	E	Control vs Myc	1.82×10^{-5}	Mann-Whitney U test
		Control vs Myc, hid[1]/+	8.74×10^{-1}	Mann-Whitney U test
		Myc vs Myc, hid[1]/+	1.71×10^{-3}	Mann-Whitney U test
S13	D	Control vs Myc	1.05×10^{-2}	Mann-Whitney U test
		Control vs Myc, hemocyte-ablated	1.36×10^{-1}	Mann-Whitney U test
		Myc vs Myc, hemocyte-ablated	3.95×10^{-3}	Mann-Whitney U test
	G	Control vs Myc	3.49×10^{-4}	Mann-Whitney U test
	H	Control vs Myc	3.49×10^{-4}	Mann-Whitney U test
S14	G	Control vs Myc	3.21×10^{-5}	Mann-Whitney U test
	J	Control vs Myc	3.95×10^{-3}	Mann-Whitney U test
	K	Actual Cas3-positive cells in/surrounding clones vs Expected Cas3-positive cells in/surrounding clones	7.04×10^{-6}	Chi-squared test
S15	D	Control HS 10min vs Myc HS 10min	1.80×10^{-2}	Mann-Whitney U test
		Myc HS 10min vs Myc HS 60min	4.87×10^{-4}	Mann-Whitney U test
S17	F	WT/WT (7.5) vs Myc/WT (7.5)	9.12×10^{-3}	Mann-Whitney U test
		WT/WT (7.5) vs WT/Myc (7.5)	8.82×10^{-1}	Mann-Whitney U test
		WT/WT (7.5) vs WT/Myc (10)	1.87×10^{-1}	Mann-Whitney U test
		WT/WT (7.5) vs WT/Myc (12.5)	1.53×10^{-3}	Mann-Whitney U test
		Myc/WT (7.5) vs WT/Myc (7.5)	3.00×10^{-2}	Mann-Whitney U test

		WT/Myc (7.5) vs WT/Myc (12.5)	$1.51x10^{-2}$	Mann-Whitney U test
	L	WT/WT vs WT/Myc	$3.27x10^{-5}$	Mann-Whitney U test
		WT/WT vs WT/Myc, hml>hid	$2.80x10^{-1}$	Mann-Whitney U test
		WT/WT vs WT/Myc, hml>egrRNAi	$6.50x10^{-1}$	Mann-Whitney U test
		WT/Myc vs WT/Myc, hml>hid	$2.99x10^{-3}$	Mann-Whitney U test
		WT/Myc vs WT/Myc, hml>egrRNAi	$2.69x10^{-4}$	Mann-Whitney U test
	P	Clone vs Clone>egrRNAi	$4.87x10^{-4}$	Mann-Whitney U test
		Clone vs Clone & hml>egrRNAi	$2.07x10^{-5}$	Mann-Whitney U test
		Clone>egrRNAi vs Clone & hml>egrRNAi	$3.33x10^{-2}$	Mann-Whitney U test
S19	D	Control vs Myc	$2.53x10^{-2}$	Mann-Whitney U test
		Control vs Myc, arm>bskRNAi	$7.82x10^{-1}$	Mann-Whitney U test
		Myc vs Myc, arm>bskRNAi	$2.70x10^{-3}$	Mann-Whitney U test
S20	F	Myc vs Myc, hml>egrRNAi(HMC)	$1.92x10^{-3}$	Mann-Whitney U test
		Myc vs Myc, hml>egrRNAi(KK)	$8.37x10^{-4}$	Mann-Whitney U test
S21	G	Myc vs Myc, bsk[1]/+	$4.72x10^{-3}$	Mann-Whitney U test
		Myc vs Myc, hml>bskRNAi	$1.27x10^{-3}$	Mann-Whitney U test
		Myc vs Myc, hml>bskDN	$1.72x10^{-3}$	Mann-Whitney U test
S22	M	Control vs Control(FRT, hml-Gal4)	$8.55x10^{-1}$	Mann-Whitney U test
		Control vs Control(FRT, UAS-hid)	$7.54x10^{-1}$	Mann-Whitney U test
		Minute vs Minute, hemocyte-ablated	$8.78x10^{-6}$	Mann-Whitney U test
		Minute vs Minute, hml>egrRNAi	$3.18x10^{-5}$	Mann-Whitney U test
	N	Minute vs	$1.74x10^{-4}$	Mann-Whitney U test

		Minute, hemocyte-ablated		
		Minute vs Minute, hml>egrRNAi	$1.62x10^{-4}$	Mann-Whitney U test
	O	Control vs Control(FRT, hml-Gal4)	$2.45x10^{-1}$	Mann-Whitney U test
		Control vs Control(FRT, UAS-hid)	$8.84x10^{-1}$	Mann-Whitney U test
		Minute vs Minute, hemocyte-ablated	$1.45x10^{-3}$	Mann-Whitney U test
		Minute vs Minute, hml>egrRNAi	$7.47x10^{-4}$	Mann-Whitney U test
	P	Minute vs Minute, hemocyte-ablated	$1.45x10^{-3}$	Mann-Whitney U test
		Minute vs Minute, hml>egrRNAi	$1.05x10^{-3}$	Mann-Whitney U test
