# Supplementary material for: Epithelial cell competition is promoted by signaling from immune cells
Source: Nat Commun. 2025 Apr 19;16:3710. doi: 10.1038/s41467-025-59130-5 (PMC12008283; doi:10.1038/s41467-025-59130-5)
Supplement: Supplementary file 2 — Description of Additional Supplementary Files [file 41467_2025_59130_MOESM2_ESM.pdf]

**Description of Additional Supplementary Files**

**Supplementary Code 1:** Mathematica code used in data analysis for Figure 2
