# Supplementary material for: Epithelial cell competition is promoted by signaling from immune cells
Source: Nat Commun. 2025 Apr 19;16:3710. doi: 10.1038/s41467-025-59130-5 (PMC12008283; doi:10.1038/s41467-025-59130-5)
Supplement: Supplementary file 3 — Supplementary Code 1 [file 41467_2025_59130_MOESM3_ESM.pdf]

Evaluating the distances between hemocytes and clones in a disc

Evaluate the observed distances between hemocytes and clones in a disc

Import files

```
GFPfile = Import["file directory"];
```

```
RFPfile = Import["file directory"];
```

```
DAPIfile = Import["file directory"];
```

Extract all boundaries of the disc

In[ ]:=
```
Binarize[GFPfile, .065];
DeleteSmallComponents[%, 250];
ColorNegate[DeleteSmallComponents[ColorNegate[%], 75]];
darkedge = MorphologicalPerimeter[%, CornerNeighbors → False]
darkedgepts = PixelValuePositions[darkedge, 1];
```

Out[ ]=

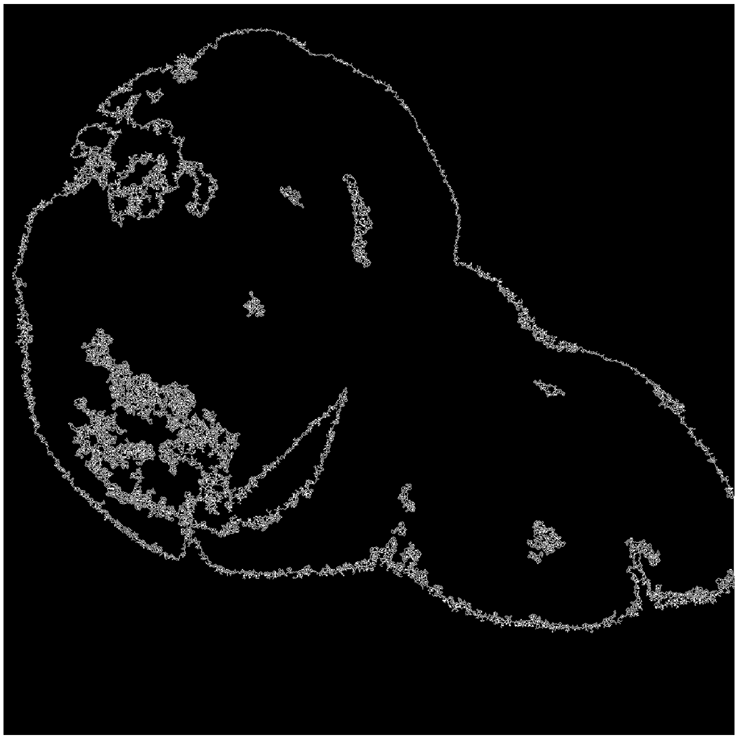

In[ ]:=
```
Binarize[GFPfile, .35];
DeleteSmallComponents[%, 250];
ColorNegate[DeleteSmallComponents[ColorNegate[%], 1000]];
brightedge = MorphologicalPerimeter[%, CornerNeighbors → False]
brightedgepts = PixelValuePositions[brightedge, 1];
```

Out[ ]=

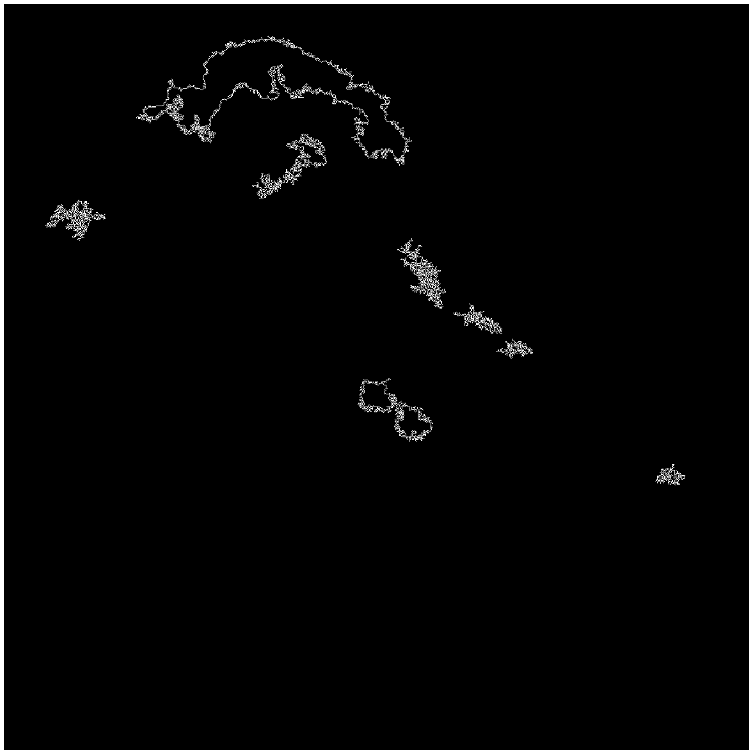

Extract the edge of the disc

In[•]:=
```
Binarize[DAPIfile, .0001];
DeleteSmallComponents[%];
ColorNegate[DeleteSmallComponents[ColorNegate[%]]];
discedge = MorphologicalPerimeter[%, CornerNeighbors → False]
discedgepts = PixelValuePositions[discedge, 1];
```

Out[•]=

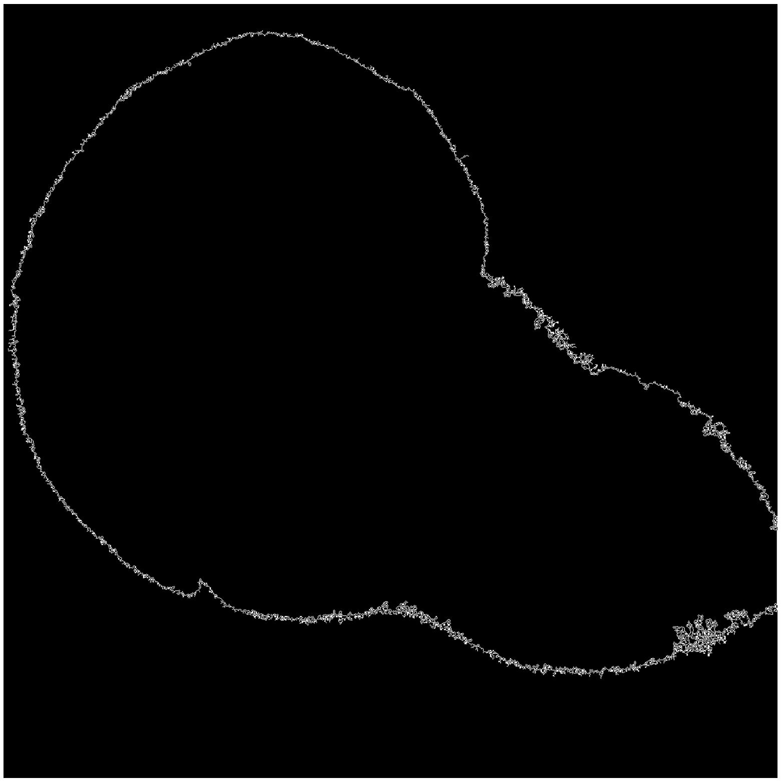

In[•]:=
```
darkedgepts' = DeleteCases[darkedgepts, Alternatives @@ discedgepts];
discedgepts' = DeleteCases[discedgepts, Alternatives @@ darkedgepts];
edgepts = Join[brightedgepts, darkedgepts', discedgepts'];
```

Extract the locations of hemocytes in the disc

In[∘]:=
```
Binarize[RFPfile, .135];
DeleteSmallComponents[%, 5];
ColorNegate[DeleteSmallComponents[ColorNegate[%], 75]];
hedge = MorphologicalPerimeter[%, CornerNeighbors → False]
hedgepts = PixelValuePositions[hedge, 1];
```

Out[∘]=

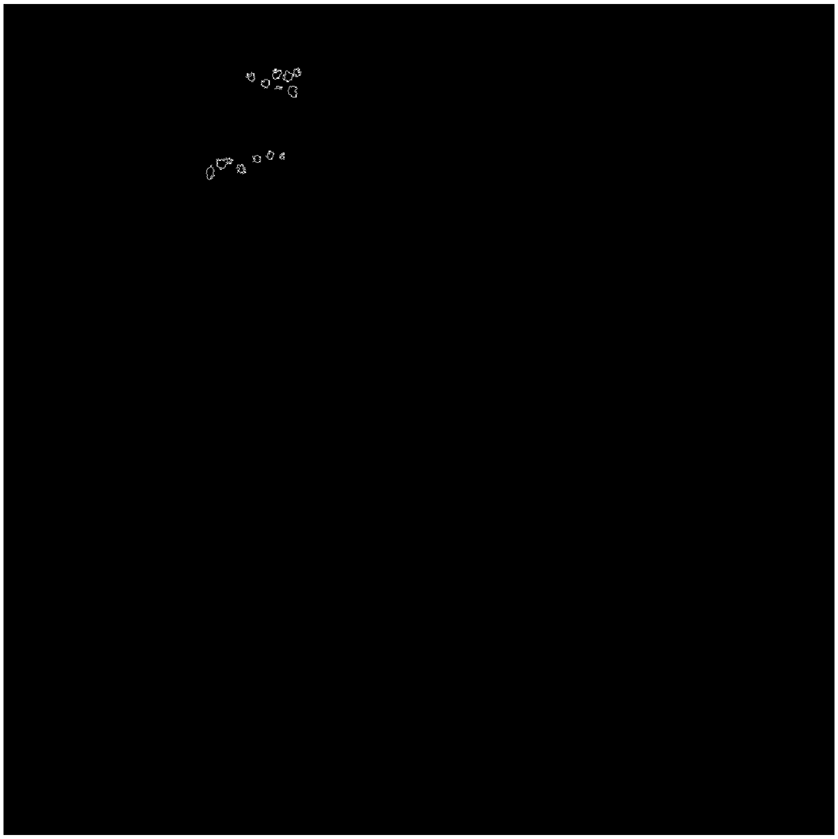

In[∘]:=
```
hemocyteCenterPts =
  {{356.3840026855469`, 916.41796875`}, {361.4132385253906`, 939.4706420898438`},
   {351.219970703125`, 934.5935668945312`}, {336.5592041015625`,
    937.319580078125`}, {339.4510498046875`, 920.9925537109375`},
   {323.6279296875`, 925.9755859375`}, {305.93048095703125`, 933.4601440429688`},
   {342.1739501953125`, 836.6297912597656`},
   {328.65362548828125`, 837.4131774902344`}, {313.6759338378906`,
    832.564697265625`}, {292.7698974609375`, 820.3772583007812`},
   {277.35980224609375`, 830.40576171875`}, {268.5753173828125`,
    827.135498046875`}, {254.42852783203125`, 817.1202392578125`}};
```

Extract boundaries of clones in the disc

In[•]:=
```
Show[ListPlot[darkedgepts', PlotStyle → Darker[Green]],
 ListPlot[discedgepts', AxesOrigin → {0, 0},
  PlotRange → {{0, 1024}, {0, 1024}}, PlotStyle → Blue], ImageSize → 1300]
```

Out[•]=

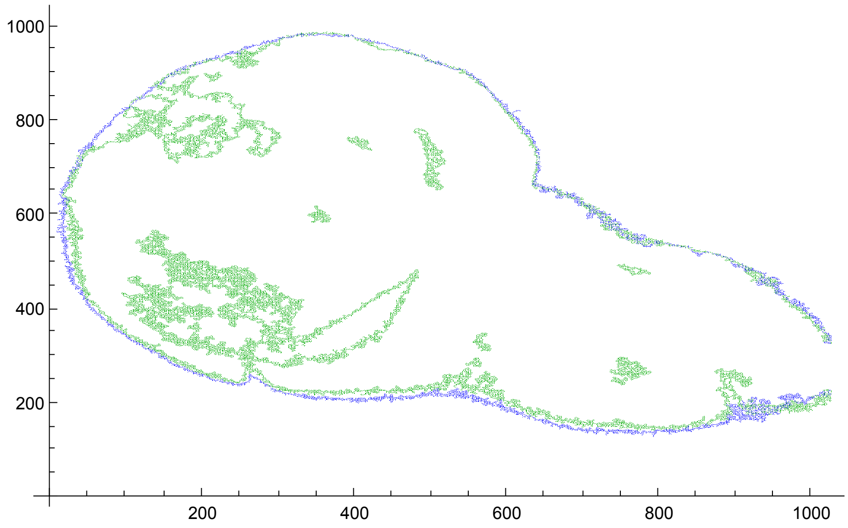

In[•]:=
```
Show[ListPlot[edgepts], ListPlot[hedgepts, AxesOrigin → {0, 0},
  PlotRange → {{0, 1024}, {0, 1024}}, PlotStyle → Red], ImageSize → 1300]
```

Out[•]=

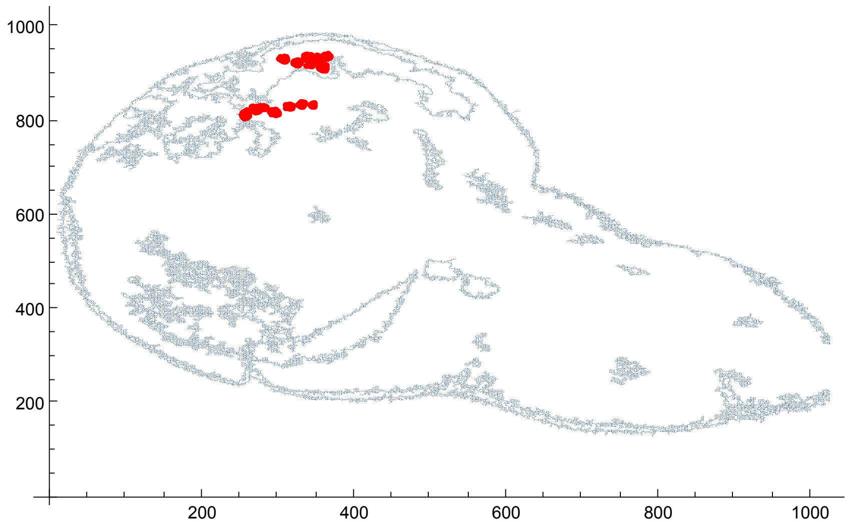

In[ ]:=
```
Show[ListPlot[darkedgepts'], ListPlot[hedgepts, AxesOrigin → {0, 0},
  PlotRange → {{0, 1024}, {0, 1024}}, PlotStyle → Red], ImageSize → 1300]
```

Out[ ]=

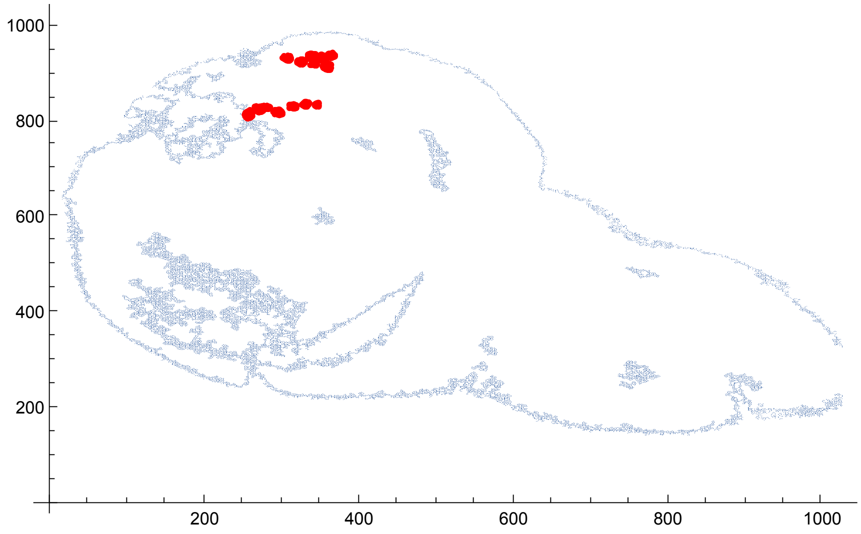

In[ ]:=
```
Show[ListPlot[brightedgepts], ListPlot[hedgepts, AxesOrigin → {0, 0},
  PlotRange → {{0, 1024}, {0, 1024}}, PlotStyle → Red], ImageSize → 1300]
```

Out[ ]=

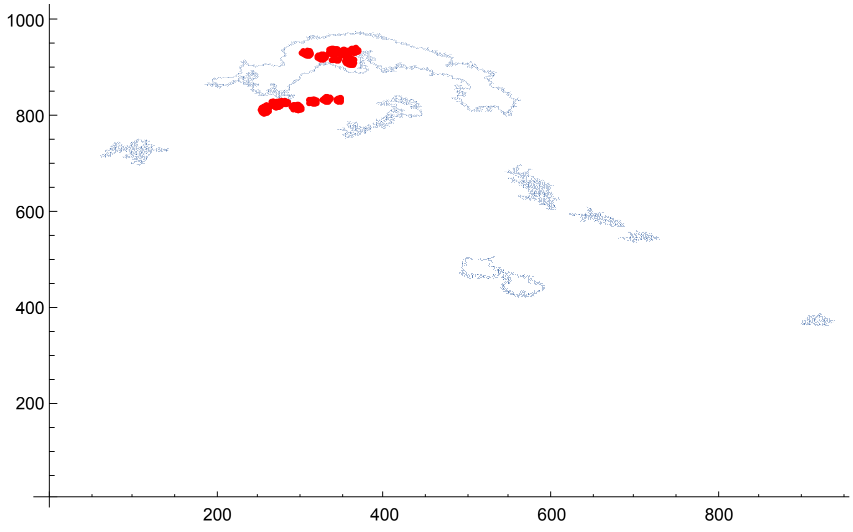

In[ ]:=
```
DarkMinDST = Table[N[EuclideanDistance[darkedgepts'[[i]],
     Nearest[discedgepts', darkedgepts'[[i]]][[1]]]], {i, 1, Length[darkedgepts']}];
DiscMinDST = Table[N[EuclideanDistance[discedgepts'[[i]],
     Nearest[darkedgepts', discedgepts'[[i]]][[1]]]], {i, 1, Length[discedgepts']}];
```

In[•]:=
```
n = 10;
Darkpos = Flatten@Position[DarkMinDST, _? (# ≥ n &)];
Discpos = Flatten@Position[DiscMinDST, _? (# ≥ n &)];
darkedgepts'' = darkedgepts'⟦Darkpos⟧;
discedgepts'' = discedgepts'⟦Discpos⟧;
Show[ListPlot[darkedgepts'', PlotStyle → Darker[Green]],
 ListPlot[discedgepts'', AxesOrigin → {0, 0},
  PlotRange → {{0, 1024}, {0, 1024}}, PlotStyle → Darker[Green]], ImageSize → 1300]
```

Out[•]=

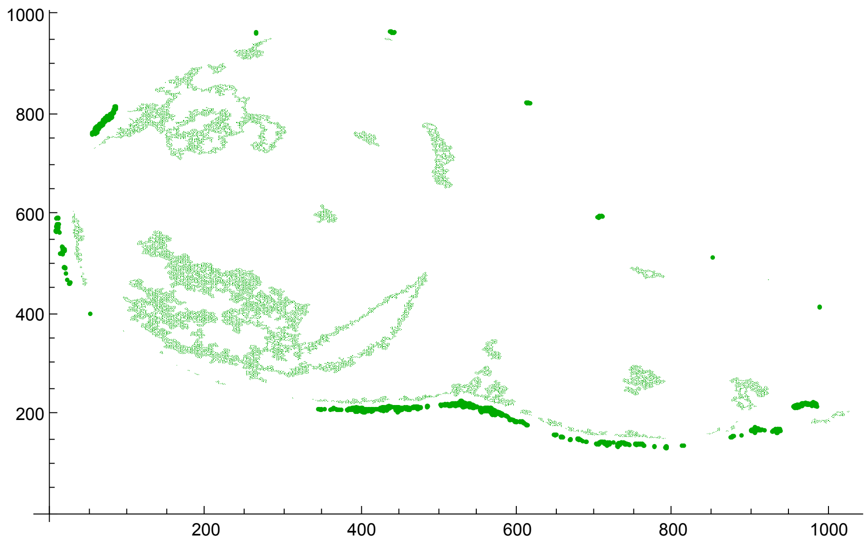

In[•]:=
```
cloneedgepts = Join[brightedgepts, darkedgepts'', discedgepts''];
```

Calculate the distances between each hemocyte and the nearest boundary

In[•]:=
```
DSTinPXwhole = Table[EuclideanDistance[hemocyteCenterPts⟦i⟧,
    Nearest[edgepts, hemocyteCenterPts⟦i⟧] // Flatten],
   {i, 1, Length[hemocyteCenterPts]}]
```

$$DSTin\mu mwhole = \frac{\% * 425.10}{1024}$$

Out[•]=

```
{11.7022, 6.57703, 12.316, 21.6146, 8.1009, 10.0695, 21.3789,
 46.1701, 38.6203, 26.0779, 14.8224, 5.16485, 10.1551, 5.54291}
```

Out[•]=

```
{4.85802, 2.73036, 5.11281, 8.97303, 3.36298, 4.18022, 8.87516,
 19.1669, 16.0327, 10.8259, 6.15334, 2.14412, 4.21577, 2.30107}
```

Calculate the distances between each hemocyte and the nearest clonal boundary

In[∘]:=
```
DSTinPXclone = Table[EuclideanDistance[hemocyteCenterPts⟦i⟧,
    Nearest[cloneedgepts, hemocyteCenterPts⟦i⟧] // Flatten],
   {i, 1, Length[hemocyteCenterPts]}]
DSTinμmclone = (% * 425.10)/1024
```

Out[∘]=
```
{11.7022, 6.57703, 12.316, 21.6146, 8.1009, 10.0695, 21.3789,
 46.1701, 38.6203, 26.0779, 14.8224, 5.16485, 10.1551, 5.54291}
```

Out[∘]=
```
{4.85802, 2.73036, 5.11281, 8.97303, 3.36298, 4.18022, 8.87516,
 19.1669, 16.0327, 10.8259, 6.15334, 2.14412, 4.21577, 2.30107}
```

Calculate the distances between each hemocyte and the nearest edge of the disc

In[∘]:=
```
DSTinPXedge = Table[EuclideanDistance[hemocyteCenterPts⟦i⟧,
    Nearest[discedgepts, hemocyteCenterPts⟦i⟧] // Flatten],
   {i, 1, Length[hemocyteCenterPts]}]
DSTinμmedge = (% * 425.10)/1024
```

Out[∘]=
```
{65.6184, 42.0485, 48.6871, 44.3297, 60.9107, 52.5139, 38.1397,
 141.467, 133.476, 129.002, 130.886, 116.616, 117.306, 120.547}
```

Out[∘]=
```
{27.2406, 17.4559, 20.2118, 18.4029, 25.2863, 21.8004, 15.8332,
 58.7283, 55.4109, 53.5537, 54.3357, 48.4114, 48.6981, 50.0434}
```

Evaluate the expected distances between hemocytes and clones

Repeat running the codes from the above section for each disc and group all data together as follows

```
DSTinPXedgeTOT = {128.71431078623866`, 130.64066939510073`, 5.383803244948671`,
    10.965371541162156`, 0.8017022604985337`, 0.36103353347805717`,
    15.665693016312893`, 44.52108457046758`, 3.460121972313312`, 3.69309079080243`,
    31.818752297295344`, 15.31437764734818`, 31.76927240024228`,
    55.662822512049814`, 93.79302690969756`, 179.10078425555523`,
    198.55026925609596`, 190.5610088185341`, 203.77037048895554`,
    231.34139663779186`, 50.977727428961245`, 176.90743194970318`,
```

```
115.172201513571`, 97.1270132260291`, 148.07907917731245`, 125.5915539195775`,
110.81090273339402`, 64.16831749327083`, 51.67245136227345`, 287.003799949269`,
282.99412136554247`, 292.5637872934199`, 307.95816787994346`,
314.8374717504939`, 70.6864898210949`, 59.87043767140306`, 52.62733174860487`,
56.87616074630981`, 28.5268408623956`, 17.43118809732383`, 30.201998012771405`,
16.619437911844273`, 2.9635835678421234`, 8.562726420816885`,
26.292431479187776`, 10.09612044072263`, 16.917795857449374`,
13.809338957786693`, 7.909361663060326`, 8.88806755252333`, 10.369154347026512`,
11.543363982823168`, 7.268740305685647`, 12.428260406041447`,
11.694747242274648`, 12.75565544193232`, 9.914693253122152`,
17.543135818787437`, 10.005040672291665`, 16.450056699593144`,
23.7864339833661`, 17.378356524721628`, 9.359826847029186`, 16.15359190580563`,
65.61836750472659`, 42.04846008064095`, 48.6870707697198`, 44.32968137941456`,
60.91073839771423`, 52.513878087024686`, 38.13969700079715`,
141.46729005831529`, 133.47618380478684`, 129.0024660352965`,
130.8862876523666`, 116.61552249403516`, 117.306128432322`, 120.54673008265719`,
2.0798879111654034`, 2.636764396387164`, 6.440025019856702`,
8.005413820106751`, 137.01468437428832`, 131.43471481862835`,
109.90566642893545`, 125.57246683895846`, 52.95267545764487`,
42.93134011314901`, 46.61547870502428`, 50.90735874767339`, 60.76430265462115`,
60.09512544658241`, 76.7831937822818`, 57.82399847517383`, 56.20859963311789`,
73.31121872728338`, 71.54117384816567`, 71.82944402695655`, 70.353843706971`,
89.0096495044666`, 89.47231452432067`, 99.14392445900378`, 90.74506791858475`,
113.59959809425447`, 120.45832055085612`, 110.38272015072518`,
98.01436810883008`, 122.10249699208688`, 133.73055592400095`,
122.59719493992868`, 108.5900942516251`, 105.69122539071293`,
132.82844147436177`, 103.49912182612944`, 129.6015691394341`,
113.76914160897081`, 107.89755355994713`, 101.64933899748704`,
121.83133170682812`, 93.33837124466639`, 39.25361391226664`,
65.51623170193847`, 61.33656654405378`, 75.30041633625287`, 66.31745935294735`,
81.37244775450502`, 63.66966200232872`, 90.89889836845111`, 81.73872748887773`,
97.39954496340333`, 70.0849113393751`, 68.13849965372621`, 100.77409764896312`,
95.70410068660773`, 83.62333800741737`, 74.51742381044738`, 65.51241214519273`,
133.75755371283503`, 126.05669343353135`, 138.60340878958178`,
124.74648174520838`, 163.73939369613157`, 156.14415074258594`,
147.69955768010678`, 135.23447846233725`, 188.46085921852756`,
182.4026347114523`, 167.74419252184182`, 160.26854450745708`,
135.59013820561742`, 148.67213238058898`, 142.87993423737913`};
```

Measure the proportions of hemocytes that fell within eight different bins that were determined by the distances away from the edge of the disc

```
Histogram[DSTinPXedgeTOT, {0, 320, 5}, "Probability", PlotRange → {0, 0.15}];
𝒟 = HistogramDistribution[DSTinPXedgeTOT, 64];
Plot[PDF[𝒟, x], {x, 0, 320}, Filling → Axis, PlotRange → All]
d = Table[RandomVariate[𝒟], 1000];
Histogram[d, 64];
Histogram[d, 6];
data = HistogramList[d, {50}, "Count"]
```

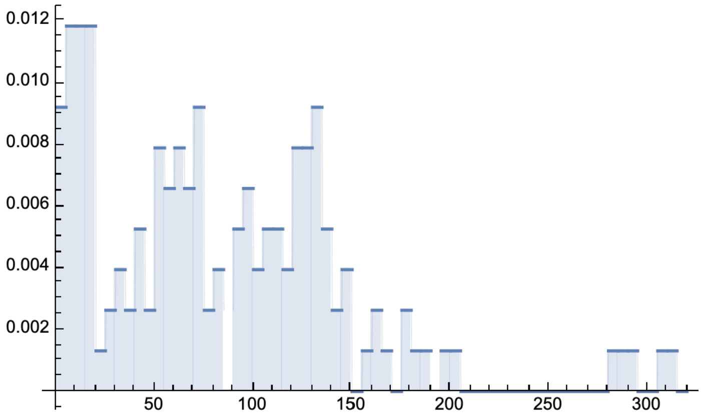


```
{{0, 50, 100, 150, 200, 250, 300, 350}, {327, 282, 279, 66, 11, 21, 14}}
```

```
In[∘]:= data = {{0, 50, 100, 150, 200, 250, 300, 350}, {327, 282, 279, 66, 11, 21, 14}};
```

Divide the disc into eight concentric territories representing the bins above

```
In[∘]:= ℛ = MeshRegion[discedgepts, Line[Table[i, {i, Length[discedgepts]}]]];
r50 = RegionUnion[
   Table[DiscretizeRegion[Disk[discedgepts⟦i⟧, 50]], {i, 1, Length[discedgepts]}]];
r100 = RegionUnion[Table[
    DiscretizeRegion[Disk[discedgepts⟦i⟧, 100]], {i, 1, Length[discedgepts]}]];
r150 = RegionUnion[Table[
    DiscretizeRegion[Disk[discedgepts⟦i⟧, 150]], {i, 1, Length[discedgepts]}]];
r200 = RegionUnion[Table[
    DiscretizeRegion[Disk[discedgepts⟦i⟧, 200]], {i, 1, Length[discedgepts]}]];
r250 = RegionUnion[Table[
    DiscretizeRegion[Disk[discedgepts⟦i⟧, 250]], {i, 1, Length[discedgepts]}]];
r300 = RegionUnion[Table[
    DiscretizeRegion[Disk[discedgepts⟦i⟧, 300]], {i, 1, Length[discedgepts]}]];
r350 = RegionUnion[Table[
    DiscretizeRegion[Disk[discedgepts⟦i⟧, 350]], {i, 1, Length[discedgepts]}]];
r400 = RegionUnion[Table[
    DiscretizeRegion[Disk[discedgepts⟦i⟧, 400]], {i, 1, Length[discedgepts]}]];
```

In[ ]:=
```
t50 = RegionDifference[ℛ, r50];
t100 = RegionDifference[t50, r100];
t150 = RegionDifference[t100, r150];
t200 = RegionDifference[t150, r200];
t250 = RegionDifference[t200, r250];
t300 = RegionDifference[t250, r300];
t350 = RegionDifference[t300, r350];
```

In[ ]:=
```
I1 = RegionIntersection[r50, ℛ];
I2 = RegionIntersection[r100, t50];
I3 = RegionIntersection[r150, t100];
I4 = RegionIntersection[r200, t150];
I5 = RegionIntersection[r250, t200];
I6 = RegionIntersection[r300, t250];
I7 = RegionIntersection[r350, t300];
I8 = RegionIntersection[r400, t350];
```

Randomly seed points into these territories proportional to the number of hemocytes that were observed in each bin

In[ ]:=
```
pts50 = RandomPoint[I1, data[[2, 1]]];
pts100 = RandomPoint[I2, data[[2, 2]]];
pts150 = RandomPoint[I3, data[[2, 3]]];
pts200 = RandomPoint[I4, data[[2, 4]]];
pts250 = RandomPoint[I5, data[[2, 5]]];
pts300 = RandomPoint[I6, data[[2, 6]]];
pts350 = RandomPoint[I7, data[[2, 7]]];
```

In[ ]:=
```
p50 = Graphics[{EdgeForm[{Black, Thick}], FaceForm[Darker[Red]], Disk[]}];
pts50plot = ListPlot[pts50, PlotMarkers → {p50, .03}];
p100 = Graphics[{EdgeForm[{Black, Thick}], FaceForm[Darker[Orange]], Disk[]}];
pts100plot = ListPlot[pts100, PlotMarkers → {p100, .03}];
p150 = Graphics[{EdgeForm[{Black, Thick}], FaceForm[Darker[Yellow]], Disk[]}];
pts150plot = ListPlot[pts150, PlotMarkers → {p150, .03}];
p200 = Graphics[{EdgeForm[{Black, Thick}], FaceForm[Darker[Green]], Disk[]}];
pts200plot = ListPlot[pts200, PlotMarkers → {p200, .03}];
p250 = Graphics[{EdgeForm[{Black, Thick}], FaceForm[Darker[Cyan]], Disk[]}];
pts250plot = ListPlot[pts250, PlotMarkers → {p250, .03}];
p300 = Graphics[{EdgeForm[{Black, Thick}], FaceForm[Darker[Blue]], Disk[]}];
pts300plot = ListPlot[pts300, PlotMarkers → {p300, .03}];
p350 = Graphics[{EdgeForm[{Black, Thick}], FaceForm[Darker[Purple]], Disk[]}];
pts350plot = ListPlot[pts350, PlotMarkers → {p350, .03}];
```

In[●]:=
```
Show[Graphics[{Red, ℛ}], Graphics[{Orange, t50}], Graphics[{Yellow, t100}],
 Graphics[{Green, t150}], Graphics[{Cyan, t200}], Graphics[{Blue, t250}],
 Graphics[{Purple, t300}], Graphics[{Pink, t350}], pts50plot,
 pts100plot, pts150plot, pts200plot, pts250plot, pts300plot, pts350plot]
```

Out[●]=

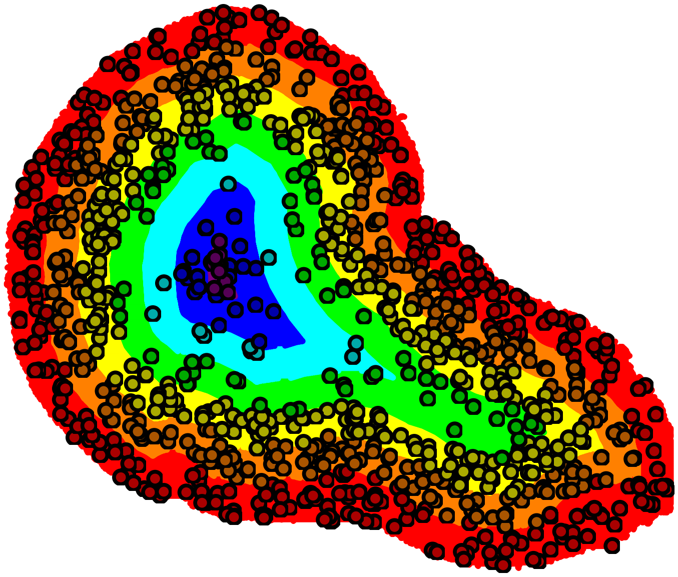

In[●]:=
```
ptstotal = Join[pts50, pts100, pts150, pts200, pts250, pts300, pts350];
Show[ListPlot[ptstotal, PlotStyle → Black], ListPlot[discedgepts],
 PlotRange → {{0, 1024}, {0, 1024}}, AxesOrigin → {0, 0}, ImageSize → Large]
```

Out[●]=

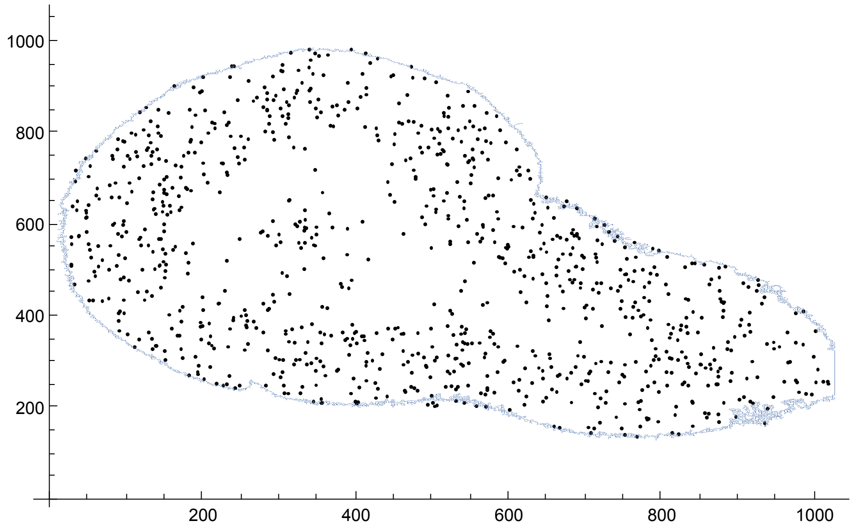

Calculate the distances between each point and the nearest boundary in the disc

```
CONDSTinPXwhole8 = Table[EuclideanDistance[ptstotal[[i]],
    Nearest[edgepts, ptstotal[[i]]][[1]]], {i, 1, Length[ptstotal]}];
CONDSTinμmwhole8 = (% * 425.10)/1024;
```

Calculate the distances between each point and the nearest clonal boundary in the disc

```
CONDSTinPXclone8 = Table[EuclideanDistance[ptstotal[[i]],
    Nearest[cloneedgepts, ptstotal[[i]]][[1]]], {i, 1, Length[ptstotal]}];
CONDSTinμmclone8 = (% * 425.10)/1024;
```

Compare the expected and observed data

```
In[ ]:= GraphicsRow[
  {Histogram[DSTinμmwhole, {0, 70, 5}, "Probability", PlotRange → {0, 1}], Histogram[
    DSTinμmclone, {0, 70, 5}, "Probability", PlotRange → {0, 1}]}, ImageSize → 1300]
Out[ ]=
```

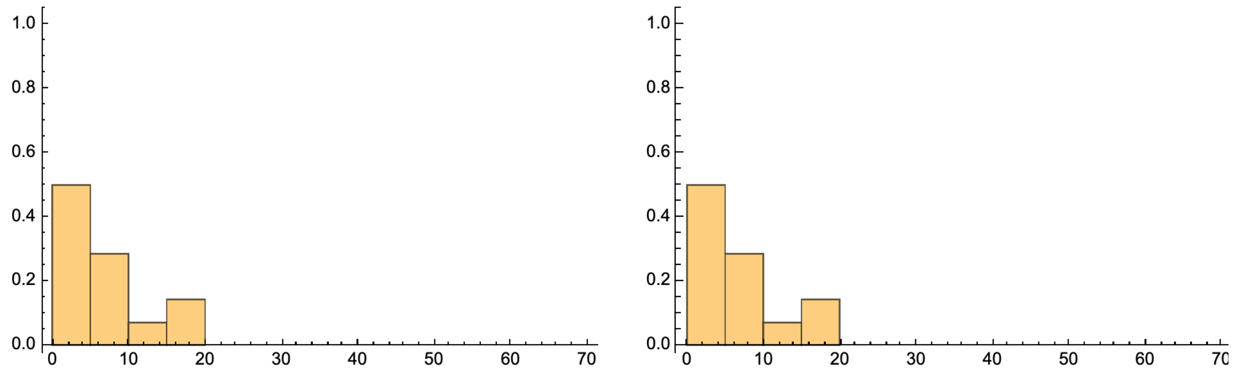

In[•]:=
```
GraphicsRow[
 {Histogram[CONDSTinμmwhole8, {0, 70, 5}, "Probability", PlotRange → {0, 1}],
  Histogram[CONDSTinμmclone8, {0, 70, 5},
   "Probability", PlotRange → {0, 1}]}, ImageSize → 1300]
```

Out[•]=

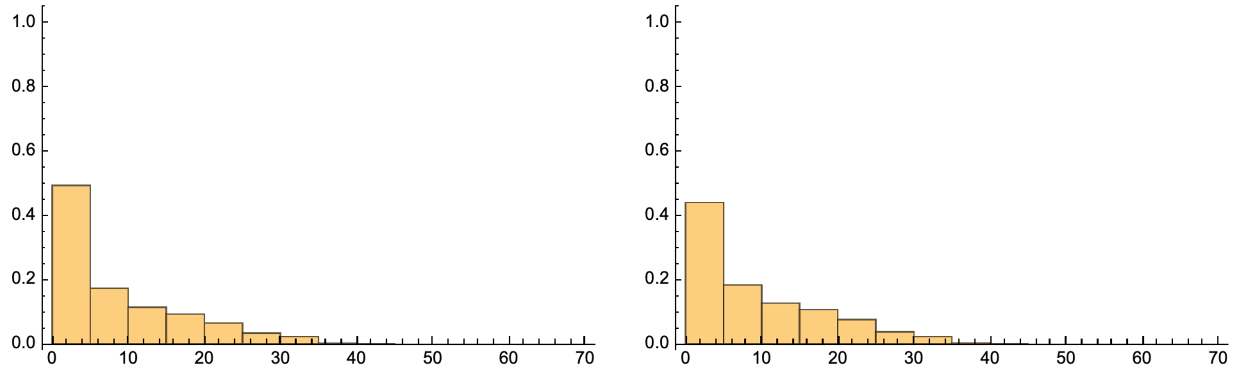

In[•]:=
```
DSTinμmwholeTOT8 = Join[DSTinμmwhole];
DSTinPXwholeTOT8 = Join[DSTinPXwhole];
DSTinμmcloneTOT8 = Join[DSTinμmclone];
DSTinPXcloneTOT8 = Join[DSTinPXclone];
```

In[•]:=
```
GraphicsRow[
 {Histogram[DSTinμmwholeTOT8, {0, 70, 5}, "Probability", PlotRange → {0, 1}],
  Histogram[DSTinμmcloneTOT8, {0, 70, 5},
   "Probability", PlotRange → {0, 1}]}, ImageSize → 1300]
```

Out[•]=

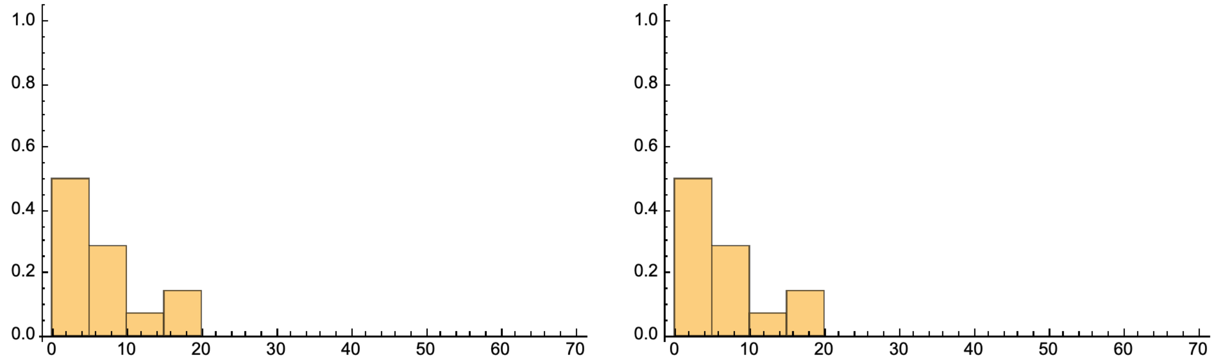

In[•]:=
```
Histogram[{CONDSTinμmwhole8}, {0, 70, 5},
 "Probability", PlotRange → {0, 1}, ChartStyle → {Red}]
```

Out[•]=

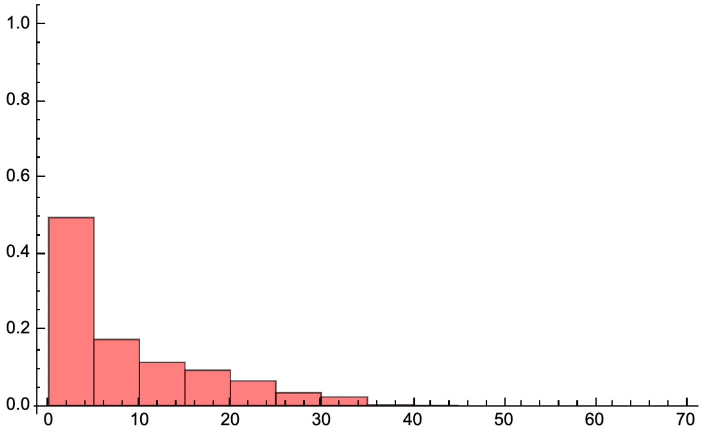

In[•]:=
```
CONDSTinμmwholeTOT8 = Join[CONDSTinμmwhole8];
CONDSTinPXwholeTOT8 = Join[CONDSTinPXwhole8];
CONDSTinμmcloneTOT8 = Join[CONDSTinμmclone8];
CONDSTinPXcloneTOT8 = Join[CONDSTinPXclone8];
```

In[•]:=
```
GraphicsRow[
 {Histogram[CONDSTinμmwholeTOT8, {0, 70, 5}, "Probability", PlotRange → {0, 1}],
  Histogram[CONDSTinμmcloneTOT8, {0, 70, 5},
   "Probability", PlotRange → {0, 1}]}, ImageSize → 1300]
```

Out[•]=

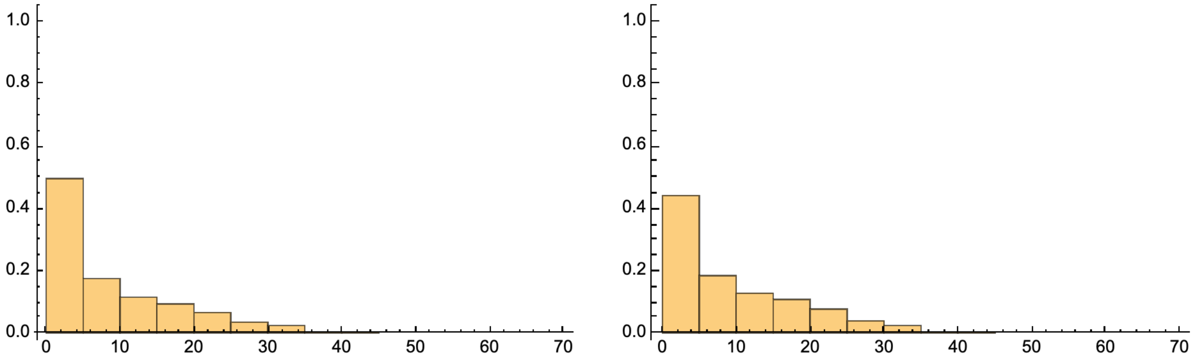

In[ ]:=
```
GraphicsRow[{SmoothHistogram[
    {DSTinμmwholeTOT8, CONDSTinμmwholeTOT8}, PlotRange → {{0, 100}, {0, .11}}],
  SmoothHistogram[{DSTinμmwholeTOT8, CONDSTinμmwholeTOT8},
   PlotRange → {{0, 100}, {0, .11}}]}, ImageSize → 1300]
```

Out[ ]=

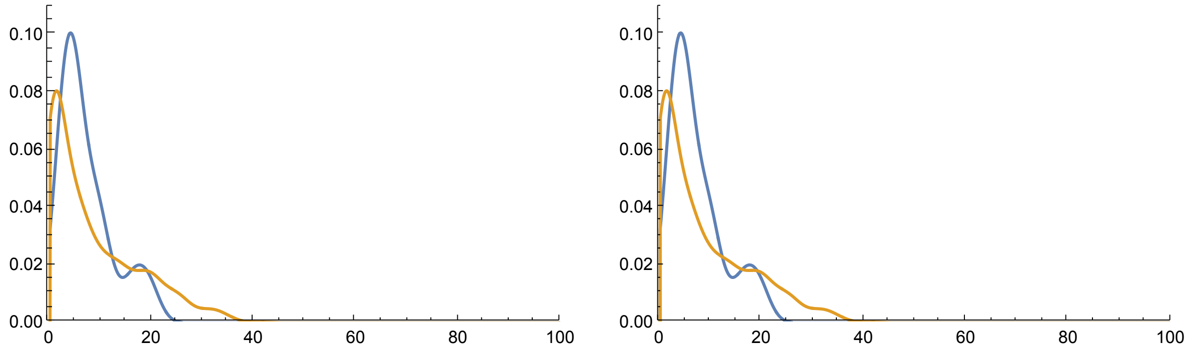

In[ ]:=
```
DSTinPXedgeTOT8 = Join[DSTinPXedge]
```

Out[ ]=
```
{65.6184, 42.0485, 48.6871, 44.3297, 60.9107, 52.5139, 38.1397,
 141.467, 133.476, 129.002, 130.886, 116.616, 117.306, 120.547}
```

In[ ]:=
```
DSTinμmedgeTOT8 = Join[DSTinμmedge]
```

Out[ ]=
```
{27.2406, 17.4559, 20.2118, 18.4029, 25.2863, 21.8004, 15.8332,
 58.7283, 55.4109, 53.5537, 54.3357, 48.4114, 48.6981, 50.0434}
```
