# Supplementary material for: Epithelial cell competition is promoted by signaling from immune cells
Source: Nat Commun. 2025 Apr 19;16:3710. doi: 10.1038/s41467-025-59130-5 (PMC12008283; doi:10.1038/s41467-025-59130-5)
Supplement: Supplementary file 4 — Reporting Summary [file 41467_2025_59130_MOESM4_ESM.pdf]

nature portfolio

Corresponding author(s): Arthur D. Lander

Last updated by author(s): Mar 28, 2025

Reporting Summary

Nature Portfolio wishes to improve the reproducibility of the work that we publish. This form provides structure for consistency and transparency in reporting. For further information on Nature Portfolio policies, see our Editorial Policies and the Editorial Policy Checklist.

Statistics

For all statistical analyses, confirm that the following items are present in the figure legend, table legend, main text, or Methods section.

n/a	Confirmed	
☐	☒	The exact sample size (*n*) for each experimental group/condition, given as a discrete number and unit of measurement
☐	☒	A statement on whether measurements were taken from distinct samples or whether the same sample was measured repeatedly
☐	☒	The statistical test(s) used AND whether they are one- or two-sided<br>*Only common tests should be described solely by name; describe more complex techniques in the Methods section.*
☐	☒	A description of all covariates tested
☐	☒	A description of any assumptions or corrections, such as tests of normality and adjustment for multiple comparisons
☐	☒	A full description of the statistical parameters including central tendency (e.g. means) or other basic estimates (e.g. regression coefficient) AND variation (e.g. standard deviation) or associated estimates of uncertainty (e.g. confidence intervals)
☐	☒	For null hypothesis testing, the test statistic (e.g. *F*, *t*, *r*) with confidence intervals, effect sizes, degrees of freedom and *P* value noted<br>*Give P values as exact values whenever suitable.*
☒	☐	For Bayesian analysis, information on the choice of priors and Markov chain Monte Carlo settings
☒	☐	For hierarchical and complex designs, identification of the appropriate level for tests and full reporting of outcomes
☒	☐	Estimates of effect sizes (e.g. Cohen's *d*, Pearson's *r*), indicating how they were calculated

Our web collection on statistics for biologists contains articles on many of the points above.

Software and code

Policy information about availability of computer code

Data collection	Software built in the microscopes were used for image acquisition.
Data analysis	Image analysis was performed using (Fiji Is Just) ImageJ (version 2.9.0/1.53t). Quantitative and statistical analysis was performed using Wolfram Mathematica 12.3.0.0 and Microsoft Excel for Mac 16.74.

For manuscripts utilizing custom algorithms or software that are central to the research but not yet described in published literature, software must be made available to editors and reviewers. We strongly encourage code deposition in a community repository (e.g. GitHub). See the Nature Portfolio guidelines for submitting code & software for further information.

Data

Policy information about availability of data

All manuscripts must include a data availability statement. This statement should provide the following information, where applicable:

- Accession codes, unique identifiers, or web links for publicly available datasets
- A description of any restrictions on data availability
- For clinical datasets or third party data, please ensure that the statement adheres to our policy

All data generated or analyzed during this study are included in this published article (and its supplementary information files).

Research involving human participants, their data, or biological material

Policy information about studies with human participants or human data. See also policy information about sex, gender (identity/presentation), and sexual orientation and race, ethnicity and racism.

Reporting on sex and gender	*Use the terms sex (biological attribute) and gender (shaped by social and cultural circumstances) carefully in order to avoid confusing both terms. Indicate if findings apply to only one sex or gender; describe whether sex and gender were considered in study design; whether sex and/or gender was determined based on self-reporting or assigned and methods used. Provide in the source data disaggregated sex and gender data, where this information has been collected, and if consent has been obtained for sharing of individual-level data; provide overall numbers in this Reporting Summary. Please state if this information has not been collected. Report sex- and gender-based analyses where performed, justify reasons for lack of sex- and gender-based analysis.*
Reporting on race, ethnicity, or other socially relevant groupings	*Please specify the socially constructed or socially relevant categorization variable(s) used in your manuscript and explain why they were used. Please note that such variables should not be used as proxies for other socially constructed/relevant variables (for example, race or ethnicity should not be used as a proxy for socioeconomic status). Provide clear definitions of the relevant terms used, how they were provided (by the participants/respondents, the researchers, or third parties), and the method(s) used to classify people into the different categories (e.g. self-report, census or administrative data, social media data, etc.) Please provide details about how you controlled for confounding variables in your analyses.*
Population characteristics	*Describe the covariate-relevant population characteristics of the human research participants (e.g. age, genotypic information, past and current diagnosis and treatment categories). If you filled out the behavioural & social sciences study design questions and have nothing to add here, write "See above."*
Recruitment	*Describe how participants were recruited. Outline any potential self-selection bias or other biases that may be present and how these are likely to impact results.*
Ethics oversight	*Identify the organization(s) that approved the study protocol.*

Note that full information on the approval of the study protocol must also be provided in the manuscript.

Field-specific reporting

Please select the one below that is the best fit for your research. If you are not sure, read the appropriate sections before making your selection.

☒ Life sciences ☐ Behavioural & social sciences ☐ Ecological, evolutionary & environmental sciences

For a reference copy of the document with all sections, see nature.com/documents/nr-reporting-summary-flat.pdf

Life sciences study design

All studies must disclose on these points even when the disclosure is negative.

Sample size	The sample sizes chosen are equal or above the typical sample sizes used in the field of developmental biology.
Data exclusions	No data exclusion
Replication	Each set of experiments was replicated independently in imaginal discs from different larvae at the same developmental stage and having the same genetic background.
Randomization	Each set of experiments was carried out on discs acquired from randomly chosen Drosophila larvae with the appropriate developmental stage and genetic background.
Blinding	Quantification was performed in the same way for all samples without being aware the genotypes.

Reporting for specific materials, systems and methods

We require information from authors about some types of materials, experimental systems and methods used in many studies. Here, indicate whether each material, system or method listed is relevant to your study. If you are not sure if a list item applies to your research, read the appropriate section before selecting a response.

Materials & experimental systems

n/a	Involved in the study	
	☒	Antibodies
☒	☐	Eukaryotic cell lines
☒	☐	Palaeontology and archaeology
	☒	Animals and other organisms
☒	☐	Clinical data
☒	☐	Dual use research of concern
☒	☐	Plants

Methods

n/a	Involved in the study	
☒	☐	ChIP-seq
☒	☐	Flow cytometry
☒	☐	MRI-based neuroimaging

Antibodies

Antibodies used

Anti-beta-Galactosidase, Purified Monoclonal Antibody (Promega Cat# Z3781, RRID:AB_430877), 1:1000
Anti-Dcp1 (Cell Signaling Technologies Cleaved Drosophila Dcp-1 (Asp215) Antibody Cat#9578,RRID: AB_2721060), 1:100
Anti-Patched (Developmental Studies Hybridoma Bank, Drosophila Ptc (Apa 1),RRID: AB_528441), 1:1000
Anti-Wingless (Developmental Studies Hybridoma Bank, 4D4, RRID: AB_528512), 1:1000
Goat anti-Mouse IgG (H+L) Cross-Adsorbed Secondary Antibody, Alexa FluorTM 555 (Thermo Fisher Scientific Cat# A-21422, RRID: AB_2535844) 1:1000
Goat anti-Rabbit IgG (H+L) Cross-Adsorbed Secondary Antibody, Alexa FluorTM 555 (Thermo Fisher Scientific Cat# A-21428, RRID: AB_2535849) 1:1000
Goat anti-Rabbit IgG (H+L) Cross-Adsorbed Secondary Antibody, Alexa FluorTM 647 (Thermo Fisher Scientific Cat# A-21244, RRID: AB_2535812) 1:1000

Validation

Anti-beta-Galactosidase (Promega Cat# Z3781, RRID:AB_430877) has been cited in more than 20 publications. Selected citations:
1. Pascual J, Jacobs J, Sansores-Garcia L, Natarajan M, Zeitlinger J, Aerts S, Halder G, Hamaratoglu F. Hippo Reprograms the Transcriptional Response to Ras Signaling. Dev Cell. 2017 Sep 25;42(6):667-680.e4. doi: 10.1016/j.devcel.2017.08.013. PMID: 28950103.
2. Zhang P, Pei C, Wang X, Xiang J, Sun BF, Cheng Y, Qi X, Marchetti M, Xu JW, Sun YP, Edgar BA, Yuan Z. A Balance of Yki/Sd Activator and E2F1/Sd Repressor Complexes Controls Cell Survival and Affects Organ Size. Dev Cell. 2017 Dec 4;43(5):603-617.e5. doi: 10.1016/j.devcel.2017.10.033. PMID: 29207260; PMCID: PMC5722641.
3. Piwko P, Vitsaki I, Livadaras I, Delidakis C. The Role of Insulators in Transgene Transvection in Drosophila. Genetics. 2019 Jun;212(2):489-508. doi: 10.1534/genetics.119.302165. Epub 2019 Apr 4. PMID: 30948430; PMCID: PMC6553826.

Anti-Dcp1 (Cell Signaling Technologies Cat#9578) has been cited in more than 300 publications. Selected citations:
1. Maurya D, Mondal BC. Larval hematopoietic organs of multiple Drosophila species show effector caspase activity and DNA damage response. MicroPubl Biol. 2024 Dec 18;2024:10.17912/micropub.biology.001392. doi: 10.17912/micropub.biology.001392. PMID: 39758584; PMCID: PMC11696351.
2. Jang S, Choi B, Lim C, Kim M, Lee JE, Lee H, Baek E, Cho KS. Neuronal fatty acid-binding protein enhances autophagy and suppresses amyloid-β pathology in a Drosophila model of Alzheimer's disease. PLoS Genet. 2024 Nov 19;20(11):e1011475. doi: 10.1371/journal.pgen.1011475. PMID: 39561115; PMCID: PMC11575808.
3. Kharrat B, Gábor E, Virág N, Sinka R, Jankovics F, Kristó I, Vilmos P, Csordás G, Honti V. Dual role for Headcase in hemocyte progenitor fate determination in Drosophila melanogaster. PLoS Genet. 2024 Oct 28;20(10):e1011448. doi: 10.1371/journal.pgen.1011448. PMID: 39466810; PMCID: PMC11515969.

Anti-Patched (Developmental Studies Hybridoma Bank, Drosophila Ptc, extracellular region (Apa 1) has been cited in 11 publications. Selected citations:
1. Zhu Y, Qiu Y, Chen W, Nie Q, Lander AD. Scaling a Dpp Morphogen Gradient through Feedback Control of Receptors and Co-receptors. Dev Cell. 2020 Jun 22;53(6):724-739.e14. doi: 10.1016/j.devcel.2020.05.029. PMID: 32574592; PMCID: PMC7437929.
2. Brás-Pereira C, Potier D, Jacobs J, Aerts S, Casares F, Janody F. dachshund Potentiates Hedgehog Signaling during Drosophila Retinogenesis. PLoS Genet. 2016 Jul 21;12(7):e1006204. doi: 10.1371/journal.pgen.1006204. PMID: 27442438; PMCID: PMC4956209.
3. Bairzin JCD, Emmons-Bell M, Hariharan IK. The Hippo pathway coactivator Yorkie can reprogram cell fates and create compartment-boundary-like interactions at clone margins. Sci Adv. 2020 Dec 9;6(50):eabe8159. doi: 10.1126/sciadv.abe8159. PMID: 33298454; PMCID: PMC7725458.

Anti-Wingless (Developmental Studies Hybridoma Bank, 4D4) has been cited in over 300 publications. Selected citations:
1. Zhu Y, Qiu Y, Chen W, Nie Q, Lander AD. Scaling a Dpp Morphogen Gradient through Feedback Control of Receptors and Co-receptors. Dev Cell. 2020 Jun 22;53(6):724-739.e14. doi: 10.1016/j.devcel.2020.05.029. PMID: 32574592; PMCID: PMC7437929.
2. Giri R, Brady S, Papadopoulos DK, Carthew RW. Single-cell Senseless protein analysis reveals metastable states during the transition to a sensory organ fate. iScience. 2022 Sep 8;25(10):105097. doi: 10.1016/j.isci.2022.105097. PMID: 36157584; PMCID: PMC9494244.
3. Kanaoka Y, Onodera K, Watanabe K, Hayashi Y, Usui T, Uemura T, Hattori Y. Inter-organ Wingless/Ror/Akt signaling regulates nutrient-dependent hyperarborization of somatosensory neurons. Elife. 2023 Jan 17;12:e79461. doi: 10.7554/eLife.79461. PMID: 36647607; PMCID: PMC9844989.

Animals and other research organisms

Policy information about studies involving animals; ARRIVE guidelines recommended for reporting animal research, and Sex and Gender in Research

Laboratory animals	Drosophila melanogaster. Experiments were performed on eye/wing discs obtained from wandering third-instar larvae. Genotypes used in this paper are given in Table S1. Drosophila melanogaster. The following strains were used in this study: arm-lacZ,M(2)Z,FRT40A (a generous gift from Jose de Celis), FRT82B,tub-HA:Mycw+ (a generous gift from Peter Gallant), y,w,hsFLP;; Act>y>Gal4,UAS-GFP.nls (X; III) (a generous gift from Hiroshi Nakato), puc-LacZ (a generous gift from Nicholas E. Baker), tub>CD2>LexA, tub>Myc>LexA, and LexOP-GFP (generous gifts from Laura A. Johnston), Act5C-Gal4 (BDSC_3954), arm-lacZ,FRT40A (BDSC_7371), arm-Gal4 (BDSC_1560), bsk1 (BDSC_3088), FRT40A (BDSC_8212), FRT82B (BDSC_86313), FRT82B,ubi-GFP (BDSC_5188), FRT82B,ubi-GFP (BDSC_32655), hid1(BDSC_631), hml-Gal4(II) (BDSC_30139), hml-Gal4(III) (BDSC_30141), hsFLP,tub>Myc>Gal4 (BDSC_64767), srpHemo-3XmCherry(II) (BDSC_78358), srpHemo-3XmCherry(III) (BDSC_78359), srpHemo-H2A.3XmCherry(II) (BDSC_78361), srpHemo-H2A.3XmCherry(III) (BDSC_78360), UAS-bskDN(X) (BDSC_6409), UAS-bskRNAi(II) (BDSC_36643), UAS-egrRNAi(II)(HMC) (BDSC_55276), UAS-egrRNAi(II)(KK) (VDRC_108814), UAS-egrRNAi(III) (VDRC_45253), UAS-hid(II) (BDSC_65403), UAS-Myc(II) (BDSC_9674), UAS-RFP(II) (BDSC_30556), UAS-RFP(III) (BDSC_31417), ubi-GFP,FRT40A (BDSC_5629).
Wild animals	the study did not involve wild animals
Reporting on sex	Since larval imaginal discs are not sexually dimorphic, both female and male larvae were randomly selected except for experiments in Figure 3 and Figure 6, for which only females were chosen as alleles on both X chromosomes were required.
Field-collected samples	the study did not involve samples collected from the field
Ethics oversight	no ethical approval or guidance was required

Note that full information on the approval of the study protocol must also be provided in the manuscript.

Plants

Seed stocks	*Report on the source of all seed stocks or other plant material used. If applicable, state the seed stock centre and catalogue number. If plant specimens were collected from the field, describe the collection location, date and sampling procedures.*
Novel plant genotypes	*Describe the methods by which all novel plant genotypes were produced. This includes those generated by transgenic approaches, gene editing, chemical/radiation-based mutagenesis and hybridization. For transgenic lines, describe the transformation method, the number of independent lines analyzed and the generation upon which experiments were performed. For gene-edited lines, describe the editor used, the endogenous sequence targeted for editing, the targeting guide RNA sequence (if applicable) and how the editor was applied.*
Authentication	*Describe any authentication procedures for each seed stock used or novel genotype generated. Describe any experiments used to assess the effect of a mutation and, where applicable, how potential secondary effects (e.g. second site T-DNA insertions, mosiacism, off-target gene editing) were examined.*
